# Abiotic stress responses in plants: roles of calmodulin-regulated proteins

**DOI:** 10.3389/fpls.2015.00809

**Published:** 2015-10-14

**Authors:** Amardeep S. Virdi, Supreet Singh, Prabhjeet Singh

**Affiliations:** ^1^Texture Analysis Laboratory, Department of Food Science & Technology, Guru Nanak Dev UniversityAmritsar, India; ^2^Plant Molecular Biology Laboratory, Department of Biotechnology, Guru Nanak Dev UniversityAmritsar, India

**Keywords:** abiotic stress, Ca^2+^, calmodulin, calmodulin-binding proteins, plants

## Abstract

Intracellular changes in calcium ions (Ca^2+^) in response to different biotic and abiotic stimuli are detected by various sensor proteins in the plant cell. Calmodulin (CaM) is one of the most extensively studied Ca^2+^-sensing proteins and has been shown to be involved in transduction of Ca^2+^ signals. After interacting with Ca^2+^, CaM undergoes conformational change and influences the activities of a diverse range of CaM-binding proteins. A number of CaM-binding proteins have also been implicated in stress responses in plants, highlighting the central role played by CaM in adaptation to adverse environmental conditions. Stress adaptation in plants is a highly complex and multigenic response. Identification and characterization of CaM-modulated proteins in relation to different abiotic stresses could, therefore, prove to be essential for a deeper understanding of the molecular mechanisms involved in abiotic stress tolerance in plants. Various studies have revealed involvement of CaM in regulation of metal ions uptake, generation of reactive oxygen species and modulation of transcription factors such as CAMTA3, GTL1, and WRKY39. Activities of several kinases and phosphatases have also been shown to be modulated by CaM, thus providing further versatility to stress-associated signal transduction pathways. The results obtained from contemporary studies are consistent with the proposed role of CaM as an integrator of different stress signaling pathways, which allows plants to maintain homeostasis between different cellular processes. In this review, we have attempted to present the current state of understanding of the role of CaM in modulating different stress-regulated proteins and its implications in augmenting abiotic stress tolerance in plants.

## Introduction

Plants, being sessile, have evolved various biochemical and metabolic processes to sense developmental, hormonal, and environmental changes under normal and stress conditions. Of the various secondary messengers, such as hydrophobic- (diacylglycerol, phosphatidylinositol, etc.) and hydrophilic molecules (Ca^2+^, cAMP, cGMP, IP3, etc.), and gases [nitric oxide (NO), carbon monoxide, etc.] in eukaryotes, the role of Ca^2+^ has been studied most extensively (Xiong et al., 2002). Different environmental, hormonal and developmental stimuli induce transient fluctuations in cytosolic Ca^2+^ ([Ca^2+^]_cyt_) levels, the frequency and amplitude of which vary according to the strength of the signal (McCormack et al., 2005). These changes in Ca^2+^ signatures are decoded by an array of Ca^2+^-binding proteins such as (i) calcium modulating protein or calmodulin (CaM), (ii) CaM-like (CML) and other EF-hand containing Ca^2+^-binding proteins, (iii) Ca^2+^-dependent protein kinases (CDPKs), and (iv) calcineurin B-like proteins (Bouché et al., 2005 and references therein). Due to the differences in the number of EF-hand motifs, different Ca^2+^-relay sensors (CaM/CMLs) show variability in their affinity to Ca^2+^ since EF-hand motifs bind to Ca^2+^ cooperatively (Babu et al., 1988; McCormack et al., 2005).

CaM, the most well-characterized Ca^2+^ sensor, is an evolutionarily conserved, acidic, heat stable, and multifunctional protein consisting of two globular domains, each with two Ca^2+^-binding EF-hand motifs (Babu et al., 1988; Rhoads and Friedberg, 1997). Although CaM lacks its own catalytic activity, binding to or chelating of Ca^2+^ causes conformational changes in the globular domains leading to interaction with the target proteins (Snedden and Fromm, 2001; McCormack et al., 2005). Using CaM-binding transcription activators (CAMTA) for mathematical modeling, Liu et al. (2015) have recently demonstrated that interaction of Ca^2+^-CaM with the target proteins results in non-linear amplification of the Ca^2+^ signals, thereby allowing greater versatility to cells in deciphering different Ca^2+^ signatures for changes in gene expression. Contrary to humans, that contain only a single CaM protein (Fischer et al., 1988), multiple forms of this protein are reported in plants (McCormack et al., 2005; Al-Quraan et al., 2010). A total of seven genes encoding four different CaMs (CaM1/CaM4, CaM2/CaM3/CaM5, CaM6, and CaM7), that share a minimum 97% identity at the primary sequence level, are observed in *Arabidopsis thaliana* (Bender and Snedden, 2013; Zhu et al., 2015 and references therein), whereas, 10 cDNAs encoding three CaM proteins have been predicted in wheat (*Triticum aestivum*; Yang et al., 1996). Similarly, rice (*Oryza sativa*) genome contains five true genes encoding two sets of CaM proteins (Boonburapong and Buaboocha, 2007). The OsCaM1 encoded by *OsCaM1-1, OsCaM1-2*, and *OsCaM1-3* in rice differs by two amino acid residues from the CaM encoded by *OsCaM2* and *OsCaM3*. Multiple genes for CaM i.e., four, six, and seven have been reported in potato (*Solanum tuberosum*), tomato (*Lycopersicon esculentum*) and tobacco (*Nicotiana tabacum*), respectively (Zhao et al., 2013). Molecular evolution of different CaMs and CMLs in plants has been reviewed extensively in a recent study (Zhu et al., 2015). Though primarily cytosolic, CaM is also localized in peroxisomes, plastids, mitochondria, the extracellular matrix, and nuclei (Jarrett et al., 1982; Roberts et al., 1983; Dauwalder et al., 1986; Ma et al., 1999; van Der Luit et al., 1999; Reddy et al., 2002; Yang and Poovaiah, 2002a), signifying versatility in its roles. Besides CaM, plants also contain CML proteins that show 16 to 75% amino acid identity with the former and are not reported in animals (McCormack et al., 2005). *Arabidopsis* genome is predicted to encode 50 CMLs that show variable number of EF hands ranging from 1 (CML1) to 6 (CML12) (McCormack et al., 2005). Recent studies have demonstrated specific roles for some of the CMLs in developmental, hormonal and stress responses (Bender and Snedden, 2013). The proteins that bind to CaM do not show conservation in their primary amino acid sequence. However, the different CaM-binding proteins (CaMBPs) are characterized by the presence of amphiphilic α-helical domains that interact with CaM through both hydrophobic and strong electrostatic interactions (O'Neil and DeGrado, 1990). Recent studies employing proteome microarray have revealed that of the 1133 *Arabidopsis* proteins tested, ~25% showed interaction with one or the other isoform of CaM/CML (Popescu et al., 2007). However, interaction of most of these proteins with CaM/CML *in vivo* awaits validation by parallel strategies such as bimolecular fluorescence complementation (BiFC) or fluorescence resonance energy transfer (FRET) assays. The targets of CaM comprise a disparate group of proteins such as metabolic enzymes, kinases, transcription factors, etc., and have been reviewed extensively (Bouché et al., 2005; Reddy et al., 2011; Das et al., 2014). Since adverse environmental conditions pose major challenge to sustainable crop productivity, therefore, our discussion in this study will focus primarily on regulation by CaM of proteins that are implicated in abiotic stress response of plants.

## Expression of CaM and its post-translational modification

The expression of genes encoding different CaM proteins in plants is affected differentially by phytohormones and environmental stresses, as was observed for *MBCaM1* and *MBCaM2* in *Vigna radiate* (Botella and Arteca, 1994). This study revealed that exogenous application of IAA and exposure to salt stress resulted in upregulation of *MBCaM1*, whereas expression of *MBCaM2* was not affected significantly. Though expression of both *AtCaM3* and *AtCaM7* was enhanced by heat shock in *A. thaliana*, the increase in transcript level of the former was observed earlier (Liu et al., 2005). Differential regulation of different *CaM* genes by diverse abiotic stress conditions can be attributed to differences in the upstream regulatory elements (Park et al., 2009; Jung et al., 2010; Chinpongpanich et al., 2012) that may enable the plants to respond in a stimulus-specific manner (Al-Quraan et al., 2010).

Post-translational methylation at Lysine-115 (L-115) is a prominent feature in most CaMs (Klee and Vanaman, 1982). L-115 methylation of CaM has been implicated in protection against adenosine triphosphate (ATP)-ubiquitin-dependent proteolysis that affects its intracellular levels (Gregori et al., 1985). Activity of target proteins can be affected differently by the CaM methylation status. This is also evident from earlier studies where activities of glutamate decarboxylase (GAD; Yun and Oh, 1998) and myosin light chain kinase in plants (Roberts et al., 1984) were shown to be independent of CaM methylation but activation of NAD kinase was adversely affected (Roberts et al., 1986; Harding et al., 1997). Role of methylation of CaM in regulating different cellular processes was further validated by overexpression of a synthetic gene encoding VU-3 CaM in transgenic tobacco plants. VU-3 CaM cannot undergo methylation due to substitution of L-115 with Arg. These transgenic plants showed impaired growth and development due to hyperactivation of a CaM-dependent NAD kinase, and consequent increase in NADPH and reactive oxygen species (ROS) levels (Harding et al., 1997). These studies provided evidence that CaM-methylation is critical for regulation of different developmental pathways in plants.

Methylation of CaM is catalyzed by CaM N-methyletransferase (CaM-KMT), a highly conserved protein among eukaryotes (Magnani et al., 2010). Expression of *CaM-KMT* gene is differentially modulated by phytohormones and abiotic stresses. Contrary to cytokinin (kinetin) that down-regulated *CaM-KMT*, the expression of this gene was shown to increase in response to auxin, salt-, and water stress (Banerjee et al., 2013), implying its role in hormone and stress signaling pathways. Overexpression of *CaM-KMT* and its suppression in *Arabidopsis*, resulting in hyper- and hypomethylation, respectively, were associated with attenuated and enhanced root length phenotype, respectively (Banerjee et al., 2013). Furthermore, the hypomethylated CaM lines also exhibited greater tolerance to abscisic acid (ABA), cold-, and salt stress, suggesting that methylated form of CaM might be interacting specifically with effector proteins of different stress signaling pathways. This conclusion was also supported by the observations that as compared to unmethylated CaM, the methylated CaM showed specifically higher binding affinity with proteins such as germin-like proteins (GLP9 and GLP10), cytochrome P450 20A1 (CP20A), and N-xylose isomerase (Banerjee et al., 2013). CaM provides further versatility in fine tuning of cellular responses to different stimuli through alteration in its subcellular localization after post-translational modification, as observed for CaM53 in petunia (*Petunia hybrida;* Rodriguez-Concepcion et al., 1999). Plants contain several CMLs (Bender and Snedden, 2013) but studies on the regulation of these proteins by methylation have not been carried out yet. Methylation of CMLs, if demonstrated, may provide further flexibility in the regulation of different developmental and stress signaling pathways in plants.

## Role of CaM in maintenance of Ca^2+^ homeostasis

In comparison to cell wall and organelles, where Ca^2+^ is in millimolar concentration, Ca^2+^ in the cytosol is maintained at relatively lower levels (100–200 nm) because higher concentration of this ion is toxic for phosphate-based energy systems (Bush, 1995; Reddy, 2001; Clapham, 2007). Intracellular levels of Ca^2+^ in cytoplasm and endomembrane system are regulated through control of influx and efflux mechanisms. Though, passive influx of Ca^2+^ into cytosol takes place through Ca^2+^-channels (Sanders et al., 1999), its efflux is an active process that is mediated by Ca^2+^/H^+^ antiporters and Ca^2+^ pumps. Energy for this process is provided by ATP hydrolysis and proton motive force (Bush, 1995). Two types of Ca^2+^-ATPases, IIA and IIB, have been reported in plants and animals (Axelson and Palmgren, 1998). The endoplasmic reticulum (ER)-type Ca^2+^-ATPases are known as type IIA Ca^2+^ pumps and activity of these pumps is not regulated by CaM (Chung et al., 2000). On the contrary, the activity of type IIB Ca^2+^ pumps is stimulated by CaM (Malmström et al., 1997; Harper et al., 1998), and these proteins are localized to tonoplast (Malmström et al., 1997), plasma membrane (PM; Bonza et al., 2000), chloroplast inner membrane (Huang et al., 1993), and ER (Hong et al., 1999).

Expression of Ca^2+^-ATPase encoding genes is differentially modulated under different stress conditions. Exposure to high salt (NaCl) concentrations and fungal elicitor was observed to enhance the expression of soybean Ca^2+^-ATPase encoding *SCA1* gene, whereas, addition of mannitol and KCl had no apparent effect (Chung et al., 2000). The SCA1 protein consists of two CaM-binding domains (CaMBDs) at amino acid residues 1–40 and 52–71, respectively, in the N-terminus region and binds to CaM in a Ca^2+^-dependent manner. The ATPase activity of SCA1 is stimulated following its interaction with Ca^2+^-CaM. The N-terminus domain is autoinhibitory for ATPase activity of SCA1 since deletion of this region resulted in activity that was similar to native protein assayed in the presence of Ca^2+^-CaM. Though Chung et al. (2000) did not study whether stress-induced increase in mRNA transcripts of *SCA1* was accompanied by an increase in the corresponding protein, it is likely that enhanced levels of this enzyme may be contributing to lowering of Ca^2+^ to the basal levels so as to prevent Ca^2+^ toxicity (Clapham, 2007). It appears that regulation of Ca^2+^ levels through Ca^2+^-pumps, which are modulated by CaM, appears to be a part of feedback mechanism that maintains Ca^2+^ homeostasis in the cell. Since canonical and divergent CaM isoforms are reported to bind differentially to one of the *Arabidopsis* Ca^2+^-ATPases (AtACA8; Luoni et al., 2006), systematic studies on the kinetics of stress-induced changes in [Ca^2+^]_cyt_, and expression of different Ca^2+^-ATPases and CaM isoforms are required to elucidate the mechanism of this feedback regulation.

## Regulation of glutamate decarboxylase by CaM

γ-Amino butyric acid (GABA) is an ubiquitously found non-protein amino acid. Characterized as a neurotransmission inhibitor of central nervous system in animals (Mody et al., 1994; Hampe et al., 2001), the role of this molecule is still a matter of speculation in plants. GABA is produced as a result of glutamate decarboxylation, catalyzed by GAD. The basic structure of animal and plant GADs is conserved (Ueno, 2000) and these proteins show 75–86% similarity at the protein sequence level (Gallego et al., 1995; Johanson et al., 1997; Turano and Fang, 1998; Yun and Oh, 1998; Akama et al., 2001; Bouché et al., 2004; Oh et al., 2005; Lee et al., 2010). The genes encoding GADs in plants are present in multiple copies, with five copies each predicted in the genomes of rice as well as *Arabidopsis* (Akama and Takaiwa, 2007). Expression of different GAD isoforms in plants is regulated in a tissue-dependent manner and is induced by different abiotic stresses such as heat-, osmotic-, and oxidative stress (Zik et al., 1998; Lee et al., 2010). These observations indicate that GADs play important roles in growth and development, and stress responses in plants. Contrary to animals and *Escherichia coli* (Ueno, 2000), GAD proteins in yeast (Coleman et al., 2001) and plants (Baum et al., 1993; Gallego et al., 1995; Turano and Fang, 1998; Zik et al., 1998; Lee et al., 2010) are characterized by the presence of a CaMBD in the proximal C-terminal region. Of the two GAD proteins in rice, OsGAD1 shows the presence of an authentic CaMBD (Akama et al., 2001), suggesting that the CaM-binding property could be isoform-dependent. The maximum activity of plant GADs is observed under acidic conditions and is independent of Ca^2+^-CaM. However, at neutral pH, Ca^2+^-CaM becomes an obligatory requirement for the activity of these proteins (Snedden et al., 1995). Stress-induced increase in GABA levels, attributed to enhanced acidification and increase in Ca^2+^ levels, leads to Ca^2+^-CaM-induced dimerization of C-terminus domains that results in activation of GADs (Arazi et al., 1995; Snedden et al., 1995; Baum et al., 1996). Overexpression of petunia *GAD* gene, that encoded a protein lacking the CaM-binding domain, in transgenic tobacco resulted in severe morphological abnormalities indicating the role of glutamate and GABA in plant growth and development (Baum et al., 1993, 1996). Constitutive overexpression of the truncated *OsGAD2*, that encoded a protein lacking the C-terminus region, resulted in 40-fold increase in its activity. This experiment is the first evidence of Ca^2+^-CaM-independent activation of GAD enzyme in plants (Akama and Takaiwa, 2007). It is evident that C-terminal domain of OsGAD2 protein is involved in autoinhibition. Although a GAD protein lacking the CaMBD (OsGAD2) has only been reported in rice, these studies suggest that regulation of GADs through Ca^2+^-CaM-dependent and Ca^2+^-CaM-independent pathways may provide greater versatility to plants in their response to different environmental conditions. Genome level surveys in different plant species might shed more light on the presence and roles of CaMBD-lacking GADs in Ca^2+^/CaM signaling pathways.

## Implications of CaM in regulation of plant responses to heavy metals and xenobiotic compounds

Intensive industrialization and agriculture processes result in the release of toxic heavy metals such as nickel, cobalt, cadmium, copper, lead, chromium, and mercury which degrade the ecosystem and are consistently threatening agricultural production, particularly in the developing countries. Toxic levels of heavy metals in plants adversely affect the protein and enzyme structures through interaction with sulfhydryl groups and disrupt the integrity of PM, thereby, inhibiting different metabolic processes such as photosynthesis and respiration (Ovečka and Takáč, 2014; Emamverdian et al., 2015). Uptake of heavy metal ions in plants is mediated through channel proteins that are localized to PM. These proteins consist of transmembrane domains and a putative cyclic nucleotide monophosphate domain that overlaps with CaMBD at C-terminus (Köhler et al., 1999). Overexpression of an 81 kDa PM-localized protein, NtCBP4, in tobacco conferred higher levels of tolerance to Ni^2+^ due to reduced uptake of this metal ion (Arazi et al., 1999). NtCBP4 protein is homologous to *Arabidopsis* cyclic nucleotide gated channel protein CNGC1 and binds to CaM. The transgenic plants, however, showed hypersensitivity to Pb^2+^ that was attributed to its over accumulation. Subsequent studies revealed that deletion of CaM- and cyclic nucleotide binding domains resulted in abrogation of Pb^2+^-hypersensitivity, primarily due to attenuation in the uptake of this ion by transgenic plants (Table 1; Sunkar et al., 2000). These studies demonstrate that alteration in CaM-binding property of the channel proteins is a potentially viable strategy for engineering tolerance to toxic metals in crop plants, hence, needs to be explored further.

**Table 1:** **Functional characterization of genes encoding different calmodulin (CaM)-binding proteins by transgenic analysis**.

**S. No**.	**CaM-binding protein**	**Source of gene**	**Transgenic host**	**Approach**	**Effect on stress tolerance**	**References**
**CaM-BINDING TRANSCRIPTION FACTORS**						
1	CaM-binding transcription activator (CAMTA3)/*A. thaliana* signal responsive 1 (AtSR1)	*Arabidopsis thaliana*	*A. thaliana*	T-DNA insertion mutants	Increase in tolerance to CS	Doherty et al., 2009; Du et al., 2009
2	60 kDa CaM-binding protein (CBP60g)	*A. thaliana*	*A. thaliana*	Constitutive overexpression	Enhancement in DS tolerance	Wan et al., 2012
3	CaM-binding trihelix transcription factor (AtGTL1)	*A. thaliana*	*A. thaliana*	Loss-of-function mutation	Improved DS tolerance	Yoo et al., 2010
4	CaM-binding-GTL1 (PtaGTL1)	*Populus tremula x Populus alb*a	*A. thaliana*	Constitutive overexpression	Decline in DS tolerance	Weng et al., 2012
5	25 kDa CaM-binding protein (AtCAMBP25)	*A. thaliana*	*A. thaliana*	Constitutive expression	Decrease in OS tolerance	Perruc et al., 2004
6	CaM-binding WRKY39 (AtWRKY39)	*A. thaliana*	*A. thaliana*	*wrky39* mutant	Impaired HS tolerance	Li et al., 2010
				Constitutive overexpression	Increase in HS tolerance	Li et al., 2010
**CaM-BINDING KINASES, PHOSPHATASES AND OTHER ENZYMES**						
7	Ca^2+^-dependent CaM receptor-like kinase (GsCBRLK)	*Glycine soja*	*A. thaliana*	Constitutive overexpression	Enhanced tolerance to CS, SS and OS	Yang et al., 2010a
8	Ca^2+^/CaM-regulated receptor-like kinase (AtCRLK1)	*A. thaliana*	*A. thaliana*	T-DNA knock-out mutation	Increase in CS tolerance	Yang et al., 2010b
9	CaM-binding protein kinase 3 (AtCBK3)	*A. thaliana*	*A. thaliana*	Constitutive overexpression	Enhanced tolerance to HS	Liu et al., 2008
10	CaM-binding Ser/Thr phosphatase (AtPP7)	*A. thaliana*	*A. thaliana*	Constitutive overexpression	Increase in HS tolerance	Liu et al., 2007
11	CaM-binding mitogen-activated protein kinase phosphatase (NtMKP1)	*N. tabacum*	*N. tabacum*	Constitutive overexpression	Reduction in wound-induced activation of genes	Yamakawa et al., 2004
12	CaM-binding MKP1 (OsMKP1)	*Oryza sativa*	*O. sativa*	*Retrotransposon Tos17*-mediated loss-of-function mutation	Constitutive activation of wound response	Katou et al., 2007
13	Full length CaM-binding protein/cyclic nucleotide gated channel (NtCBP4)	*Nicotiana tabacum*	*N. tabacum*	Constitutive overexpression	Enhanced tolerance to Ni^2+^ but hypersensitivity to Pb^2+^	Arazi et al., 1999
	Truncated NtCBP4 lacking CaMBD	*N. tabacum*	*N. tabacum*	Constitutive overexpression	Abrogation of Pb^2+^-hypersensitivity	Arazi et al., 1999
14	Apyrase (PsNTP9)	*Pisum sativam*	*A. thaliana*	Constitutive overexpression	Increased tolerance to cyclohexane and *N*^6^-[2-isopentyl]adenine (2iP)	Windsor et al., 2003

*CaMBD, CaM-binding domain; CS, cold stress; DS, drought stress; HS, heat stress; MAPK, mitogen-activated protein kinase; OS, osmotic stress*.

Ca^2+^-CaM pathway is also implicated in the regulation of apyrases in plants. These proteins hydrolyze nucleoside di- and triphosphates. Hydrolysis of these nucleosides in animals has been demonstrated to be essential for neurotransmission (Todorov et al., 1997) and in prevention of thrombosis (Marcus et al., 1997). In plants, activity of a pea (*Pisum sativum*) apyrase, PsNTP9, localized to nucleus and also present extracellularly (Thomas et al., 2000), was shown to be enhanced by Ca^2+^-CaM (Chen and Roux, 1986). Subsequent studies revealed that different isoforms of apyrases in *Arabidopsis* bind differentially to CaM (Steinebrunner et al., 2000). Of the two different apyrases AtAPY1 and ATAPY2 (*A. thaliana* apyrase 1 and 2) in *Arabidopsis*, only the former showed interaction with CaM. The differential regulation of different isoforms by Ca^2+^-CaM may imply distinct role of these proteins in plants that warrants further elucidation. Overexpression of pea apyrase, besides resulting in enhanced growth and phosphate transport in transgenic plants (Thomas et al., 2000), is also reported to confer higher level of tolerance to different herbicides (Windsor et al., 2003) and toxic concentrations of cyclohexane and plant growth regulators (Thomas et al., 2000; Table 1). How these physiological attributes are modulated through Ca^2+^-CaM pathway remains unknown and need to be investigated experimentally by overexpressing apyrases that lack different domains.

Exposure to plants to high salt concentration is associated with accumulation of cytotoxic compound methylglyoxal, that is produced as a byproduct of glycolysis metabolism (Yadav et al., 2005). Methylglyoxal, present in low concentration (μM) in all organisms studied (Richard, 1993), is detoxified by the enzymes glyoxalase I (GlyI) and glyoxalase II (GlyII; Mannervik and Ridderström, 1993). Expression of *GlyI* and *GlyII* genes in plants is enhanced under different abiotic stress conditions (Kaur et al., 2014a,b and references therein). Overexpression of *Gly* genes from wheat (*TaGlyI), Beta vulgaris* (*BvM14*-*GlyI*), *Brassica juncea* (*GlyI*) and rice (*GlyII)* has been reported to confer enhanced tolerance to salt-, metal-, osmotic- and oxidative stress in transgenic plants (Singla-Pareek et al., 2003, 2006; Lin et al., 2010; Alvarez Viveros et al., 2013; Wu et al., 2013), implying the role of these genes in stress adaptation.

Deswal and Sopory (1999) demonstrated that activity of GlyI enzyme was stimulated by CaM, Ca^2+^ and Mg^2+^ ions, with CaM-induced increase being additive when both the ions were present together. However, binding of CaM with GlyI protein *in vivo*, and regulation of glyoxalase pathway by Ca^2+^-CaM needs to be demonstrated by using BiFC/FRET assays, mutants and pharmacological approaches. Recent studies have revealed up to 11 different *Gly* genes in rice and *A. thaliana* (Kaur et al., 2013). Since no information is available on CaMBD(s) in members of GlyI and GlyII families, we carried out *in silico* analysis of these proteins from rice and *Arabidopsis* using online tool (http://calcium.uhnres.utoronto.ca/ctdb/ctdb/browse.html). This study revealed the presence of up to three different putative CaMBDs in several members of GlyI and GlyII protein families in *Arabidopsis* and rice (Table S1). AtGlyI-13.5 and OsGlyI-1 proteins also showed the presence of an unclassified CaMBD and an IQ motif, respectively. Different *GlyI* genes in rice show differential inducibility under different abiotic stress conditions (Mustafiz et al., 2011). Therefore, specificity of CaM for different Gly proteins need to be investigated for understanding the role of Ca^2+^/CaM pathway in the regulation of these enzymes.

## Regulation of reactive oxygen species by CaM

Production of ROS is one of the major determinants of stress-induced damage to the cells (Mittler, 2002). Hydrogen peroxide (H_2_O_2_), one of the ROS, is a secondary signal molecule that constitutes an important component of signal transduction pathways that enable the plants to respond to changes in external stimuli (Del Río, 2015). H_2_O_2_ in plants is generated during photorespiration, mitochondrial electron transport and β-oxidation of fatty acids (Scandalios et al., 1997), and its intracellular levels are stringently controlled for maintaining homeostasis in the cell. Catalase degrades H_2_O_2_ into water and O_2_, and is one of the critical antioxidant enzymes that protect the cells against ROS-induced damage under stressful conditions. As compared to single isoform in animals, plants contain multiple isoforms of catalases (McClung, 1997; Scandalios et al., 1997). Contrary to bacteria, bovine, human or fungi, plants also contain catalases that are activated after binding to Ca^2+^-CaM (Yang and Poovaiah, 2002b). The CaMBD of *Arabidopsis* catalase, AtCAT3, is located in the C-terminus amino acid residues 415–451 and is autoinhibitory for its enzyme activity (Yang and Poovaiah, 2002b). It has been postulated that binding of Ca^2+^-CaM to this domain relieves autoinhibition and makes these proteins catalytically active in response to Ca^2+^ spike. For studying variability in CaMBDs of different catalases, we carried out multiple sequence alignment that revealed high homology in this region [73.0 and 70.3% identity of AtCAT3 CaMBD with rice and sorghum (*Sorghum bicolor*) homologs, respectively] (Figure S1; Table S2), suggesting functional conservation.

H_2_O_2_ is also reported to activate Ca^2+^-channels (Pei et al., 2000), thereby, inducing an increase in the [Ca^2+^]_cyt_ levels (Price et al., 1994). Paradoxically, the production of H_2_O_2_ from NADPH is catalyzed by NADPH oxidase that requires continuous influx of Ca^2+^ for its activity (Keller et al., 1998). Furthermore, the activity of NAD kinase that generates the substrate (NADPH) for NADPH oxidase from NAD^+^ is also modulated through Ca^2+^-CaM (Harding et al., 1997). These observations indicate toward an intricate mechanism of feedback regulation of different enzymes through Ca^2+^ and CaM that may enable the plants to maintain H_2_O_2_ homeostasis, thus offering protection against stress-induced damage.

## CaM is involved in modulation of transcription factors

Precise regulation of gene expression is imperative for successful completion of life cycle of an organism and its capability to withstand adverse environmental conditions. Expression of genes is regulated by a battery of transcription factors (TFs) and approximately two- and three-thousand genes encoding these proteins have been identified in human and *A. thaliana*, respectively (The *Arabidopsis* Genome Initiative, 2000). Activity of several TFs is modulated by Ca^2+^ (Bouché et al., 2002), either by direct binding, as for DREAM proteins (Carrión et al., 1999) or through Ca^2+^-CaM. Modulation of TFs by CaM can either be through direct interaction, as observed for helix-loop-helix TFs (Corneliussen et al., 1994) or through the control of kinase-mediated phosphorylation (Corcoran and Means, 2001). The proteins belonging to CaM-binding transcription activator (CAMTA) family of TFs in plants are conserved at structural and sequence levels, suggesting their essential role in the cell (Choi et al., 2005). The expression of different *CAMTA* genes is induced in response to different environmental cues such as heat stress, light, hormone, and pathogen attack (Yang and Poovaiah, 2002a), implying their role in stress response. Multiple isoforms of CAMTA proteins are reported in plants. Six genes (*AtCAMTA1-6*) encoding different proteins have been identified in *Arabidopsis* (Bouché et al., 2002; Finkler et al., 2007), rice (Choi et al., 2005), and sorghum (this study; Table S3). CAMTA proteins are characterized by the presence of specific domains that are responsible for nuclear localization (a bipartite signal in the N-terminal), non-sequence-specific DNA-binding domain (TIG domain), protein-protein interaction domain (ankyrin domain) and IQ domain in the C-terminus (Gly^872^–Arg^889^; Bouché et al., 2002).

A rice CaM-binding TF, OsCBT, that shows 44.1% amino acid identity with *Arabidopsis* AtCAMTA5 (Table S3) was demonstrated to possess two CaMBDs, CaMBD1 (amino acid residues 764–777) and CaMBD2 (amino acid residues 825–845; Figure S2; Table S4; Choi et al., 2005). CaMBD1, consisting of an IQ-motif, binds to CaM in a Ca^2+^-independent manner and constitutes the Ca^2+^-dependent CaM-dissociation domain. On the contrary, interaction of CaMBD2 with CaM is Ca^2+^-dependent. Therefore, the presence of these domains enables OsCBT to interact with CaM in absence as well as presence of Ca^2+^. OsCBT is localized to nucleus due to the presence of two different nuclear localization signals (NLSs) at both N-terminus (amino acid residues 73–90) and C-terminus (amino acid residues 837–840; Choi et al., 2005). Studies by these authors further revealed that coexpression of *OsCaM* and *OsCBT* genes resulted in inhibition of transcriptional activity of the latter, thereby providing evidence for the role of CaM in regulation of gene expression.

In order to understand the significance of CaM-regulation of CAMTA proteins in plants, we retrieved amino acid sequences of these proteins from *Arabidopsis*, rice, and sorghum genome databases and carried out multiple sequence alignment analysis of CAMBD1 and CAMBD2 (Figure S2; Table S5). This analysis revealed that Ca^2+^-dependent CaMBD2 is conserved among these proteins, whereas CaMBD1 is observed only in AtCAMTA5 and 6 in *Arabidopsis*, and in a single isoform in sorghum (acc. no. XP_002462876.1; Figure S2). It is evident that in contrast to CaMBD1, CaMBD2 appears to be evolutionarily conserved in plant taxa. Conservation of Ca^2+^-dependent CaMBD (CaMBD2) in plants may be the result of an adaptive strategy to regulate the cellular responses under conditions that lead to an alteration in Ca^2+^ signal. On the contrary, the presence of Ca^2+^-dependent CaM-dissociation domain in an isoform-specific manner suggests that different members of CAMTA proteins family may be playing distinct roles in Ca^2+^/CaM pathway, thereby providing versatility to plants in responding to different environmental conditions. We also speculate that since OsCBT shows 98.7% identity to another CAMTA protein in rice (acc. no. LOC_Os07G30774.1; Table S3), the two may be polymorphic variants of the same protein that needs to be validated by analyzing additional genotypes. Understanding the functional significance of OsCBT polymorphism may reveal novel insights into the role of these proteins in Ca^2+^/CaM signaling pathway.

The C-repeat binding factors or CBF transcription factors, induced rapidly under cold stress, play an important role in acquisition of tolerance to cold stress through regulation of ~100 other genes (collectively designated as CBF-regulon) that are implicated in cold acclimation (Maruyama et al., 2004). Imposition of cold stress also results in the elevation of [Ca^2+^]_cyt_ levels due to its release from vacuolar as well as extracellular stores (Knight et al., 1996). One of the CAMTA proteins, CAMTA3, in *Arabidopsis* (also termed as *A. thaliana* signal responsive 1 or AtSR1) was demonstrated to regulate expression of CBF2 by binding to a conserved DNA-binding motif CM2 or CG-element (vCGCGb) present in the *ZAT12* promoter region of the latter (Doherty et al., 2009). These studies established a link between cold-induced increase in intracellular Ca^2+^, expression of CBF-regulon, and cold tolerance (Table 1). The role of CAMTA TFs in cold stress tolerance of plants is further supported by their localization to nucleus (Bouché et al., 2002) and by the presence of at least one CAMTA3-interacting CG-element in the promotor regions of 30 different genes induced in early stage of cold stress (Doherty et al., 2009). CAMTA3 regulates rapid and transient general stress response by binding to a *cis*-acting element RSRE (rapid stress-response element; CGCGTT; Benn et al., 2014). Though CAMTA protein is constitutively present in the nucleus and binds to DNA even in the absence of CaM (Bouché et al., 2002; Yang and Poovaiah, 2002a), the RSRE-mediated expression of genes is observed only under stress. It has been proposed that RSRE-mediated expression under stress by CAMTA3 may be induced after binding with Ca^2+^-CaM that facilitates interaction of this protein with other TFs, resulting in transcription of stress-responsive genes (Bjornson et al., 2014).

**Figure 1:**
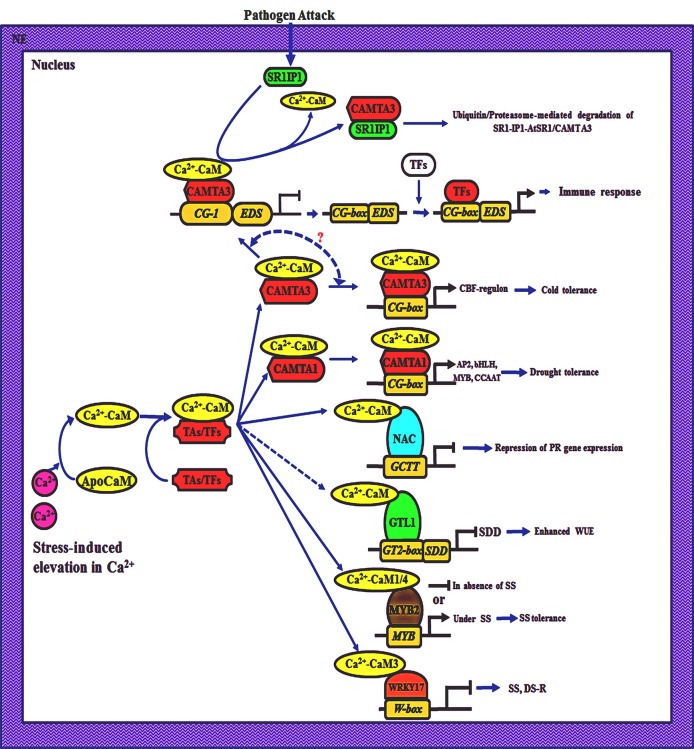
**Schematic representation of Ca^2+^/calmodulin (CaM)-mediated transcriptional regulation in plants**. Plants respond to different hormonal, developmental and environmental cues through transient fluctuations in cytosolic Ca^2+^ which is detected by CaM, leading to conformational changes in the latter. The Ca^2+^-loaded CaM (Ca^2+^-CaM) interacts with transcriptional activators (TAs) such as CaM-binding transcriptional activators (CAMTA) or transcription factors (TFs) such as NAC, MYB, WRKY, GTL1, resulting in repression (**–**⊣) or induction (→) of the downstream genes. NE, nuclear envelop; SR1IP1, SR1-Interacting-Protein-1; EDS1, Enhanced Disease Susceptibility 1; SDD1, Stomatal Density and Distribution 1; SS, Salt stress; DR, drought stress-regulon; WUE, water use efficiency; PR, Pathogenesis-Related genes.

Besides cold stress adaptation, CAMTA3 is also involved in repression of plant immunity since the *Arabidopsis* plants mutated in *CAMTA3 (atsr1)* showed enhanced resistance to virulent strain of *Pseudomonas syringae*. The *atsr1* plants depicted early induction of *PR1* (*PATHOGENESIS RELATED 1*) gene, after 6 h inoculation of *P. syringae* as compared with 24 h in wild type plants (Du et al., 2009). Repression of defense response by *AtSR1* is attributed to the suppression of *EDS1 (ENHANCED DISEASE SUSCEPTIBILITY 1)* gene that encodes a positive regulator of salicylic acid synthesis. Du et al. (2009) provided evidence that regulation of *EDS1* by AtSR1 protein is through its interaction with CG-element present in the promotor of the former. Ca^2+^ signaling is crucial for stimulating the production of ROS and NO that lead to the induction of hypersensitive response (Guo et al., 2003; Lecourieux et al., 2006; Ali et al., 2007). However, the uncontrolled defense response also affects the growth of plants adversely (Gurr and Rushton, 2005). By acting as a suppressor of immune response, *AtSR1* may be playing a central role in fine tuning the defense response of plants. Suppression of immune response by AtSR1 is regulated through Ca^2+^-CaM since deletion of CaMBD from this protein results in failure to suppress plant immunity (Du et al., 2009). The negative regulation of immune response by Ca^2+^-CaM-AtSR1 complex is proposed to be released through AtSR1-Interacting-Protein-1 (SR1-1P1) that binds to AtSR1 and facilitates ubiquitination and degradation of the latter upon pathogen challenge (Zhang et al., 2014). Though AtCaMTA3 is acting as a positive regulator of cold stress tolerance and also as a suppressor of immune response in plants, the underlying mechanism(s) responsible for cross-talk between the two pathways are not understood (Figure 1). Therefore, interaction between the pathogen-induced hyperresponse and cold acclimation needs to be elucidated by studying the kinetics of *SR1-1P1* and *AtSR1* induction under combined exposure. These studies may lead to an understanding of the different mechanisms that allow plants to maintain a balance between the two responses (Figure 1).

One of the CAMTA proteins in *Arabidopsis*, CAMTA1, has also been demonstrated to play a role in auxin response (Galon et al., 2010) and drought adaptation (Pandey et al., 2013; Figure 1). Exogenous application of auxin results in enhanced levels of [Ca^2+^]_cyt_ and induces TFs such as MYB77 that binds to auxin response factor, thereby regulating growth and development processes in plants (Shin et al., 2007; Galon et al., 2010). Galon et al. (2010) observed that expression of several genes (~17), that are otherwise induced by auxin signaling pathways, was upregulated in plants mutated in *CAMTA1*. On the contrary, expression of genes that are involved in flavonoid biosynthesis and sulfur metabolism pathways was down regulated in *CAMTA1* mutants. Expression of *CAMTA1* is reported to enhance under different stress conditions (Yang and Poovaiah, 2002a). Therefore, suppression of auxin response could be a crucial factor in maintaining homoeostasis, as inhibition of growth and development under stress may enable the plants to divert resources toward stress adaptation. Although intracellular Ca^2+^ levels are enhanced in response to both auxin and stress conditions (Galon et al., 2010), the role of Ca^2+^/CaM in *CAMTA1*-induced inhibition of auxin response has not been demonstrated. Therefore, studies employing mutants, Ca^2+^-channel blockers and CaM-antagonists are required to provide further insights into the role of Ca^2+^/CaM signaling pathway in CAMTA1-mediated auxin response.

Plants contain another large family of TFs that are characterized by the presence of NAC domain (No Apical Meristem in Petunia; *A*TAF1, ATAF2, and *C*up-Shaped Cotyledon in *Arabidopsis*), with 100–150 members reported in rice (Nuruzzaman et al., 2010) and foxtail millet (*Setaria italica*; Puranik et al., 2013). *NAC* genes have been reported to play a crucial role in stress adaptation since overexpression of one of the genes of this family, *SNAC1* (*STRESS-RESPONSIVE NAC 1*), in rice imparted drought tolerance without any associated yield penalty (Hu et al., 2006). NAC proteins are characterized by the presence of a conserved N-terminus DNA-binding domain and a C-terminus variable region that is implicated in activation (Tran et al., 2004) as well as repression (Kim et al., 2007) of the transcription. The transcription repressor activity of an *Arabidopsis* NAC protein (CBNAC) was enhanced after Ca^2+^-dependent interaction with CaM (Kim et al., 2007; Figure 1). Conserved domain analysis of different NAC proteins of *Arabidopsis*, carried out in the present study (data not shown), revealed that CaMBD (comprising of amino acid residues 471–512 in CBNAC) is not conserved among different members of this family. The selective presence of CaMBD among NAC proteins suggests that different members of this family are regulated differentially by CaM and perform distinct regulatory functions. Experimental analysis is required to determine the CaM-binding property of different NAC proteins so that the role of these proteins can be established in Ca^2+^/CaM signal transduction pathway.

Ca^2+^/CaM pathway is also implicated in the regulation of MYB TFs. These proteins regulate several aspects of growth and development such as cell cycle, morphogenesis, and secondary metabolism in plants (Kranz et al., 1998). DNA-binding activity of one of the MYB proteins, AtMYB2, that controls expression of dehydration- and salt stress-responsive genes in *Arabidopsis* was reported to enhance after interaction with a salt stress-induced isoform of *G. max* CaM (GmCaM4; Yoo et al., 2005). These authors also observed that Ca^2+^-dependent interaction of AtMYB2 with GmCaM4 resulted in transcriptional activation of genes of proline biosynthetic pathway leading to an increase in proline accumulation. On the contrary, the activity of AtMYB2 was inhibited by another CaM isoform, GmCAM1, thus, implying differential role of CaM isoforms in regulation of these TFs. Site-directed mutagenesis, resulting in substitution of Lys^69^ to Arg^69^ in CaMBD (amino acid residues 63–82) of AtMYB2 protein, abrogated the binding with GmCaM1. On the contrary, the interaction of AtMYB2 with GmCaM4 was inhibited only when both L^69^ and I^78^ were replaced with Arg (Yoo et al., 2005). These two isoforms have been proposed to play specific roles in plants. The constitutive expression of GmCaM1 maintains the transcription of AtMYB2-regulated genes at basal levels under control conditions. On the other hand, the salt stress-induced accumulation of GmCaM4 protein leads to enhanced expression of genes such as *DELTA1-PYRROLINE-5-CARBOXYLATE SYNTHASE 1, DEHYDRATION-RESPONSIVE PROTEIN RD22*, and *ALCOHOL DEHYDROGENASE 1* that encode protective proteins, leading to stress tolerance (Yoo et al., 2005). Role of *GmCaM4* in stress adaptation was also supported by the studies which demonstrated that overexpression of this gene resulted in a concomitant increase in stress tolerance of transgenic *Arabidopsis* plants, whereas overexpression of *GmCaM1* had no significant effect. Identification of other members of MYB TF family that are regulated by Ca^2+^-CaM will further enhance our understanding of the role of these proteins in signal transduction pathways responsible for stress adaptation in plants.

The WRKY transcription factors, comprising of 74 members in *Arabidopsis*, are characterized by the presence of DNA-binding or WRKY domain at N-terminus and C2H-C/H zinc motif (Eulgem et al., 2000). The WRKY domain consists of amino acid residues WRKYGOK, and based on the number of these domains and zinc motifs these TFs are grouped as G1, G2 (G2_a+b_; G2_c_; G2_d_) and G3 (Eulgem et al., 2000). One of the group G2_d_ members in *Arabidopsis*, AtWRKY7, is induced by pathogen attack and salicylic acid, and depicts interaction with CaM only in the presence of Ca^2+^ (Park et al., 2005). The CaMBD (^72^VAVNSFKKVISLLGRSR^88^) present in the C-terminus of AtWRKY7 is distinct from the classical CAMBDs described until now and is conserved in several group G2_d_ members (WRKY11, 15, 17, 21, 39, and 74; Park et al., 2005). Exposure to pathogens and salicylic acid also results in Ca^2+^ spike (Du et al., 2009). Therefore, the possibility that AtWRKY7 may be regulated through Ca^2+^-CaM pathway cannot be ruled out and needs to be demonstrated experimentally. Until recently, the WRKY TFs were implicated in the modulation of immune response, and growth and development of plants (Rushton et al., 2010). Recent studies have shown that these proteins also play an important role in abiotic stress adaptation (Tripathi et al., 2014 and references therein). A WRKY gene, *GhWRKY17*, in cotton has been implicated in drought and salt tolerance through regulation of ROS production and ABA signaling pathway. *In silico* analysis of amino acid sequences revealed that AtWRKY17 is 42.7% identical to GhWRKY17 but CaMBDs of these proteins share 78% identity, signifying functional conservation (Yan et al., 2014). However, the role of Ca^2+^-CaM pathway in regulation of activity of the GhWRKY17 and implications thereof on stress tolerance of plants are still a matter of conjecture and warrant further investigations (Figure 1).

Another plant-specific family of TFs that bind to CaM consists of CBP60 proteins (Bouché et al., 2005). One of the members of this family, CBP60g, in *Arabidopsis* showed Ca^2+^-dependent interaction with CaM (Wang et al., 2009b). Though CBP60g, localized to nucleus, is implicated in defense signaling, recent studies have shown a role of this protein in abiotic stress tolerance also (Table 1). Wan et al. (2012) observed that CBP60g-overexpressing transgenic *Arabidopsis* plants, besides exhibiting higher levels of salicylic acid and enhanced resistance to *P. syringae*, also demonstrated increased tolerance to drought stress. These authors also reported higher sensitivity of the transgenic lines to ABA. However, the molecular mechanism(s) responsible for CBP60g-induced drought tolerance in the transgenic plants and the role of Ca^2+^/CaM in its regulation are not known and need to be explored further. Apart from that, whether the ABA-hypersensitivity of transgenic plants was due to an increase in endogenous ABA or because of changes in the expression of proteins of ABA signaling pathway also needs to be addressed for deciphering the molecular basis of these observations. To conclude, these studies suggest that regulation of different TFs through Ca^2+^/CaM pathway is a crucial aspect of abiotic stress response in plants and unraveling of these mechanisms may lead to novel strategies for developing stress tolerance in different crops.

One of the most desirable features for drought stress adaptation in plants is to maximize water use efficiency (WUE; Nobel, 1999). Of the several variables affecting WUE, stomatal aperture and density, which regulate the rate of transpiration (Chaerle et al., 2005), are affected by several environmental factors such as light, temperature, CO_2_, and water availability. The family of trihelix transcription factors or GT factors, comprising of 30 and 31 members in *Arabidopsis* and rice, respectively (Riechmann et al., 2000; Wang et al., 2014b), is characterized by the presence of highly conserved trihelix domain and binds specifically to the GT-elements (Dehesh et al., 1992; Zhou, 1999). The GT factors, divided into five different groups viz., SIP1, SH4, GTγ, GT-1, and GT-2 in *Arabidopsis*, are implicated in the regulation of different developmental processes and responses to abiotic and biotic stresses (Wang et al., 2014b). Recent studies have demonstrated that one of the members of this family, *GTL1* (*GT-2 LIKE 1*), acts as a negative regulator of WUE since loss of function mutation in this gene in *Arabidopsis* resulted in enhanced tolerance to water stress (Table 1; Yoo et al., 2010). On the contrary, overexpression of its poplar (*Populus tremula* × *Populus alb*a) homolog *PtaGTL1* suppressed the drought tolerance (Weng et al., 2012; Table 1). The negative regulation of WUE by GTL1 protein is mediated through suppression of *SDD1* (*STOMATAL DENSITY AND DISTRIBUTION 1*) following its binding to GT2-box (GGAAT) in the promoter of the latter (Figure 1). *SDD1* encodes a subtilisin-like protease and represses the stomatal development through activation of genes encoding ER (ERECTA) and TMM (Too Many Mouths) proteins (von Groll et al., 2002; Shpak et al., 2005), resulting in a decrease in stomatal density and consequently enhancement in WUE. PtaGTL1 has been recently demonstrated to interact with CaM in a Ca^2+^-dependent manner through the C-terminus amino acid residues 528–551 and 555–575 (Weng et al., 2012), suggesting a role for Ca^2+^-CaM pathway in the regulation of this protein. However, effect of Ca^2+^-CaM on interaction of GTL1 protein with *SDD1* promoter, imperative for understanding the regulation of GTL1 through Ca^2+^-CaM pathway under stress conditions, has not been demonstrated *in vitro* or *in vivo* and awaits validation. GT-1 *cis*-element (GAAAAA) that binds to *Arabidopsis* GT-1-like transcription factor, AtGT-3b, is also observed in the promoter region of a soybean CaM gene, *SCaM-4* (Park et al., 2004). These authors observed that there was a concomitant and rapid increase in mRNA transcripts of *SCaM-4* and *AtGT-3b* after pathogen attack and NaCl treatment, the induction of former being attributed to the presence of GT-1 *cis*-element. Regulation of *SCaM-4* expression through interaction between *GT-1 cis*-element and GTL-1 transcription factor (Park et al., 2004) points toward a complex feedback loop that may allow precise control over Ca^2+^-CaM-mediated stress response(s). However, *in silico* and empirical analysis of promoter regions of different *CaM* genes for the presence of GT-1 elements and their regulation by GTL TFs in different plant species is required to validate this speculation.

Negative regulation of stress tolerance is also observed for a plant-specific gene encoding a 25 kDa protein, AtCAMBP25, in *Arabidopsis* that shows low affinity interaction with CaM in the presence of Ca^2+^ (Perruc et al., 2004). Interaction of AtCAMBP25 with CaM appears to be isoform-specific since it showed binding to typical CaM, AtCaM1, but not to the less conserved form, AtCaM8. AtCAMBP25 is localized to nucleus but its role in transcription is yet to be demonstrated. Although mRNA transcripts corresponding to *AtCAMBP25* showed rapid accumulation under different stress conditions, constitutive expression of this gene in transgenic lines, however, resulted in hypersensitivity to salt- and osmotic stress (Perruc et al., 2004). By virtue of being a negative regulator, it is likely that *AtCAMBP25* is involved in the maintenance of homeostasis under salt- and osmotic stress conditions (Table 1). Also, the isoform-specific interaction of CaM with this protein may enable the plants to fine tune their response in a stress-specific manner.

## Implications of Ca^2+^-CaM in signal transduction through modulation of stress-regulated kinases

Transduction of signal through phosphorylation and dephosphorylation, mediated by kinases and phosphatases, respectively, allows the cells to respond and adapt to the environmental changes (Charpenteau et al., 2004). CaM regulates the activity of several kinases in plants, and genes for these proteins have been cloned and characterized in several plant species (Zhang and Lu, 2003 and references therein). The characteristic features of different CaM-binding kinases (CBKs) include the presence of CaMBD, variable N- and C-terminal domains, and a protein kinase catalytic domain (Zhang and Lu, 2003). Expression of several CBK genes is modulated differentially by different stressors and phytohormones, suggesting their role in abiotic stress response in plants. Hua et al. (2004) reported significant increase in mRNA transcript levels of *NtCBK2* in tobacco in response to salt stress and gibberellic acid, whereas, auxin, ABA, heat-, cold-, and osmotic stress had no significant effect. These authors attributed the salt stress-induced increase in the expression of *NtCBK2* to a decrease in osmotic potential, which appears unlikely since expression of this gene was unaltered by polyethylene glycol in the same study. Therefore, it is likely that *NtCBK2* plays a role in signal transduction specifically under salt stress.

The substrate phosphorylation activity of NtCBK2, and autophosphorylation activity of *Arabidopsis* CBK, AtCBK1, were enhanced several folds after binding to Ca^2+^-CaM (Hua et al., 2003; Xie et al., 2003; Zhang and Lu, 2003; Ma et al., 2004). On the contrary, the autophosphorylation activity of lily (*Lilium longiflorum*) and tobacco CBKs was downregulated by CaM (Liu et al., 1998; Sathyanarayanan et al., 2001). It is evident that CaM acts as both negative and positive regulator of CBK activity in plants. Further, it was observed that CaM had no effect on autophosphorylation activity of maize (*Zea mays*) CBK (ZmCaMK) but its substrate phosphorylation activity showed obligated requirement for Ca^2+^-CaM (Pandey and Sopory, 1998, 2001) suggesting that different activities of the same protein can also be affected differently by CaM. The auto- and substrate phosphorylation activities of rice CBK (OsCBK), despite its higher affinity for CaM, are not regulated through Ca^2+^-CaM (Zhang et al., 2002), indicating that CaM might also be involved in modulation of these proteins through other mechanisms that are yet to be identified. Species-dependent differential regulation of CBKs by CaM signifies diversity in the perception of signals and their transduction in response to different stimuli in plant taxa.

A large family of kinases belonging to receptor-like serine/threonine kinases (RLKs), with at least 600 RLK homologs predicted in *Arabidopsis* (Hardie, 1999), is also reported in plants. Majority (75%) of the RLKs are localized to PM with the remaining (25%) present in the cytoplasm. The cytoplasmic- and PM-localized RLKs also differ in their domain architecture, with the former containing only the kinase domain (Yang et al., 2004), whereas the latter also show the presence of additional extracellular ligand-binding- and membrane spanning domains (Torii, 2000). These proteins enable the plants to respond to external cues through signal transduction by recognition of extracellular signals, followed by autophosphorylation on the cytoplasmic kinase domain (Stone and Walker, 1995). Members of both PM- and cytoplasm-localized RLK families are reported to interact with CaM and have been implicated in stress adaptation response. The transcript level of *CRCK1* that encodes CaM-binding receptor-like cytoplasmic kinase (CRCK1), a cytoplasmic RLK in *Arabidopsis*, was upregulated by salt- and cold stress, ABA, and H_2_O_2_ treatments (Yang et al., 2004). The substrate- and autophosphorylation activities of CRCK1 were enhanced following Ca^2+^-dependent interaction with CaM through amino acid residues 160–183. The kinase activities of PM-localized RLKs of *Glycine soja* (GsCBLRK; Yang et al., 2010b) and *Arabidopsis* CRLK1 (Yang et al., 2010a), implicated in salt- and cold stress tolerance, respectively, are also regulated through Ca^2+^-dependent interaction with CaM (Table 1). Compared with a single CaMBD in GsCBLRK, two CaMBDs (CaMBD1 at N-terminus amino acid residues 30–49 and CaMBD2 at C-terminus amino acid residues 369–390) are observed in CRLK1, suggesting that besides kinase activity, CaM may also be regulating other functions of this protein. This speculation is also supported by the fact that autophosphorylation activity of another PM-localized RLK, AtCaMRLK, is not affected by its interaction with Ca^2+^-CaM (Charpenteau et al., 2004). Though dynamics of interaction of GsCBLRK and CRLK1 with CaM has not been investigated, it is likely that difference in the number of CAMBDs in these two proteins results in differential affinity with CaM that may be critical for regulating their activities differentially. Variability in regulation of different activities of plant CBKs by CaM signify evolutionary divergence and is possibly the result of myriad adaptive processes operating under diverse environmental conditions that enable the plants to respond in a stimulus-specific manner.

Growth and development in plants, and their responses to stressful conditions are also modulated through mitogen-activated protein kinases (MAPKs), a different class of kinases that have been reviewed earlier (Pedley and Martin, 2005). The MAPKs are activated and inactivated through phosphorylation and dephosphorylation by MAPK kinase (MEK) and MAPK phosphatases (MKPs), respectively (Katou et al., 2007). Regulation of MKPs through CaM is a feature unique to plants and constitutes an important regulatory point in signal transduction (Katou et al., 2007). Genes coding for MKPs have been cloned and characterized from diverse plant species such as rice (*OsMKP1*; Katou et al., 2007), *Arabidopsis* (*AtMKP1*; Lee et al., 2008), wheat (*TMKP1*; Ghorbel et al., 2015), and tobacco (*NtMKP1*; Yamakawa et al., 2004; Figure S3). These proteins have been demonstrated to interact with CaM in a Ca^2+^-dependent manner. Despite high similarity in their amino acid sequences, these proteins show variability in the number of CaMBDs and their affinities toward CaM. Only a single putative CaMBD is observed in NtMKP1 and OsMKP1, compared with two in AtMKP1 (CaMBD1 at amino acid residues 445–469 and CaMBD2 at amino acid residues 669–692) and TMKP1 (CaMBD1 at amino acid residues 398–449 and CaMBD2 at amino acid residues 618–669; Lee et al., 2008; Ghorbel et al., 2015). In AtMKP1, the interaction of CaM was reported to be stronger with CaMBD2 as compared to CaMBD1. *In silico* analysis of different MKP1 proteins revealed that as compared to 40.7–59.8% in dicots, identity among monocots ranged between 68.6 and 80.5%, suggesting higher level of conservation in the latter (Table S6). The CAMBD2 in one of the maize MKP1 isoforms (ZmMKP1.2) showed less conservation as compared to CAMBD1 (Table S7). Recent studies demonstrated that deletion of the two CaMBDs (amino acid residues 398–449 and 618–669) from TMKP1, an ortholog of AtMKP1, resulted in a 4-fold increase in phosphatase activity of this protein (Ghorbel et al., 2015), suggesting an autoinhibitory role of these domains. Contrary to AtMKP1, which showed enhanced phosphatase activity following interaction with Ca^2+^-CaM (Lee et al., 2008), the effect of Ca^2+^-CaM on activity of TMKP1 was cofactor-dependent. The phosphatase activity of TMKP1 was regulated positively by Ca^2+^-CaM in the presence of Mn^2+^ or Mg^2+^, whereas interaction with Ca^2+^-CaM in the absence of these metal ions abrogated the enzyme activity (Ghorbel et al., 2015). However, Ca^2+^-CaM had no significant effect on phosphatase activity of the protein that lacked C-terminus amino acid residues. The divergence in CaMBDs and their differential affinity toward Ca^2+^-CaM may allow the plants to respond in a signal-specific manner, suggesting distinct regulatory functions of these proteins in different species. The MKPs are also proposed to act as negative regulators of defense response, as overexpression of NtMKP1 was reported to result in the suppression of kinase activity of several MAPKs that are induced in defense and wound responses (Yamakawa et al., 2004; Table 1). These observations indicate that Ca^2+^-CaM-regulated MKPs may constitute a crucial link between Ca^2+^ signaling and MAPK signaling pathways, enabling the plants to maintain homeostasis under biotic and abiotic stress conditions.

## Regulation of heat shock response through Ca^2+^/CaM signal transduction pathway

Global warming is projected to result in an increase in average temperature (Angilletta, 2009), implying that heat stress may become one of the major limiting factors for crop productivity. Recent studies suggest that yields of rice decline by 10 percent for every 1°C increase over mean minimum temperature during the growing season (Peng et al., 2004). Imposition of heat stress results in oxidative damage to cell wall, protein misfolding and denaturation or aggregation at cellular levels (Wang et al., 2004). Understanding the heat stress response in plants is, therefore, imperative for developing crops that are tolerant to high temperature stress. The plants, in general, respond to heat stress by selective repression and induction of genes. Synthesis of heat shock proteins (HSPs) is one of the protective strategies that enable the plants to cope with heat stress. The HSPs act as chaperones, prevent aggregation and recycle the aggregated proteins (Wang et al., 2004; Yamada et al., 2007). Plant HSPs are categorized into different categories viz., small HSPs or sHSPs (12–40 kDa), HSP60 (chaperonin), HSP70, HSP90 and HSP100 (Wang et al., 2004 and references therein). The promoter regions of HSP genes consist of heat shock elements (HSEs; 5′-AGAAnnTTCT-3′), that are recognized by heat shock factors (HSFs) which regulate the expression of these genes (Baniwal et al., 2004; Gao et al., 2008). As compared to other eukaryotes, the number of genes encoding HSFs in plants is substantially higher, with 21 and 25 genes reported in *Arabidopsis* and rice, respectively (Nover et al., 2001; Wang et al., 2009a).

Imposition of heat stress leads to elevation in [Ca^2+^]_cyt_, and Ca^2+^/CaM signal transduction pathway plays a crucial role in regulating the response of plants to thermal stress (Gong et al., 1998; Liu et al., 2003; Wu et al., 2012). Changes in PM fluidity in response to heat shock result in transduction of signal through cytoskeleton, Ca^2+^ signatures and CDPKs, followed by an activation of mitogen-activated protein kinases (MAPKs; Sangwan et al., 2002). Ca^2+^ has also been implicated in increased DNA-binding activity of HSFs through direct interaction (Mosser et al., 1990; Li et al., 2004). These studies, therefore, point toward involvement of Ca^2+^ in multiple regulatory pathways for controlling the heat shock response in plants. The role of Ca^2+^ in heat shock response has been further validated by the use of Ca^2+^-channel blockers, which provided evidence in favor of heat stress-induced influx of Ca^2+^ from the apoplast (Bush, 1995; Liu et al., 2003; Wu et al., 2012).

**Figure 2:**
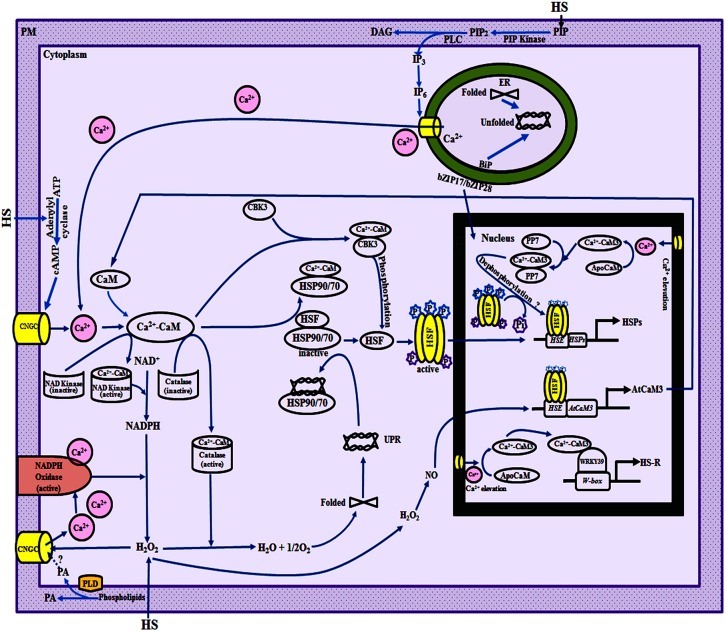
**A model illustrating the role of Ca^2+^/calmodulin (CaM) in regulation of heat shock response and H_2_O_2_ homeostasis in plants**. Changes in plasma membrane (PM) fluidity due to heat stress result in activation of phospholipase D (PLD) and phosphatidylinositol-4-phosphate 5-kinase (PIP kinase), and consequently the accumulation of various lipid signaling molecules such as phosphatidic acid (PA) and phosphatidylinositol-4, 5-bisphosphate (PIP_2_; Mishkind et al., 2009). Thermal stress also activates phospholipase C which converts PIP_2_ into diacyl glycerol (DAG) and D-myo-inositol-1,4,5-trisphosphate (IP_3_). IP_3_ may be phosphorylated and converted into IP_6_ that interacts with endoplasmic reticulum (ER)-localized Ca^2+^-channels thus resulting in release of Ca^2+^ from intracellular stores (Mishkind et al., 2009; Mittler et al., 2012). Rapid influx of extracellular Ca^2+^ in the cell can also occur due to temperature-induced activation of the PM-localized cyclic nucleotide gated channels (CNGCs) which are non-selective inward cation channels. The CNGC may be activated by heat stress-induced rapid burst in H_2_O_2_ levels (Pei et al., 2000) by cyclic adenosine monophosphate (cAMP) that is produced by heat stress-activated adenylyl cyclase (Köhler et al., 1999) and/or by PA (Mittler et al., 2012). The thermal stress-induced increase in [Ca^2+^]_cyt_ leads to conversion of ApoCaM to Ca^2+^-CaM. The expression of HSP genes is repressed in the absence of heat stress which is proposed to be due to interaction of heat shock factors (HSFs) with HSP90 (Zou et al., 1998) and/or HSP70 (Sun et al., 2000). The Ca^2+^-CaM and the denatured proteins, produced as a result of unfolded protein response (UPR) due to reactive oxygen species (ROS)-mediated oxidation, bind to HSP70/HSP90, thereby releasing the HSFs (Yamada and Nishimura, 2008; Virdi et al., 2009, 2011). H_2_O_2_ acts upstream of NO which regulates the expression of *AtCaM3* through modulation of the binding of HSF to the heat shock elements (HSEs; Wang et al., 2014a). The temperature-induced increase in NO leads to enhanced levels of *AtCaM3*, that then binds to Ca^2+^ and activates the protein kinase AtCBK3 (*Arabidopsis thaliana* Ca^2+^-CaM-binding Kinase 3), resulting in phosphorylation and trimerization of HSFs which are then translocated to the nucleus (Queitsch et al., 2000). The interaction of activated HSFs with HSEs leads to synthesis of HSPs. Dephosphorylation of the HSFs at selected amino acid residues by a nuclear-localized Ca^2+^-CaM-binding phosphatase (PP7) may lead to continuous activation of heat shock-regulon (HSR) but the precise mechanism is still not understood. The heat shock response through Ca^2+^-CaM-mediated regulation of WRKY transcription factors appears to be independent of HSF-mediated pathway. The WRKY39, after binding to Ca^2+^-CaM, interacts with the W-box elements present in the upstream promotor regions of different genes involved in thermotolerance. The ER also plays a critical role in thermal adaptation through UPR-induced release of ER membrane-tethered transcription factors such as bZIP17/bZIP28/bZIP60, which after release are translocated to the nucleus and activate the transcription of genes encoding ER-chaperones and brassinosteroid-signaling pathway related genes (Che et al., 2010; Deng et al., 2011). The intracellular levels of H_2_O_2_ under stress also appear to be maintained through Ca^2+^-CaM (Wang et al., 2014a). The H_2_O_2_-induced activation of CNGCs (Pei et al., 2000) results in an increase in the [Ca^2+^]_cyt_ (Price et al., 1994) that further activates NADPH oxidase that converts NADPH to H_2_O_2_ (Keller et al., 1998). The conversion of NAD^+^ to NADPH is catalyzed by NAD kinase that is also modulated through Ca^2+^-CaM (Harding et al., 1997). These observations, therefore, suggest the presence of an intricate feedback regulation through Ca^2+^/CaM pathway which allows the plants to maintain H_2_O_2_ homeostasis. BiP, binding immunoglobulin protein; ER, endoplasmic reticulum; HS, heat stress; NE, nuclear envelop; PM, plasma membrane.

In the absence of heat stress, HSPs in the cell are maintained at basal levels through repression of transcription of genes. Heat stress-induced increase in [Ca^2+^]_cyt_ in plants, reported to occur within 4 and 7 min in wheat and rice, respectively, precedes the induction of *CaM* genes (Liu et al., 2003; Wu et al., 2012), followed by transcriptional activation of genes that encode different HSPs. These studies, therefore, point toward the role of Ca^2+^/CaM pathway in the regulation of HSPs. Wu et al. (2012) recently reported that heat stress-induced increase in [Ca^2+^]_cyt_, and expression of *OsCaM1-1* and *OsHSP17* in rice was abrogated by CaM antagonists, chlorpromazine and trifluoperazine. Although, this aspect needs to be validated independently, these findings suggest that Ca^2+^ homeostasis and expression of *CaM* genes may be under the control of feedback regulation (Figure 2).

Recent studies in *Arabidopsis* have provided evidence that H_2_O_2_ acts upstream of NO (Wang et al., 2014a). NO regulates the expression of *AtCaM3* and functions upstream to Ca^2+^-CaM in the signal transduction pathway under heat stress (Xuan et al., 2010). The heat stress-induced increase in NO leads to enhanced levels of AtCaM3 protein that then binds to protein kinase AtCBK3 in the presence of Ca^2+^ (Liu et al., 2008), leading to phosphorylation of HSFs (Figure 2). The phosphorylated HSFs, after interaction with HSEs, activate the expression of HSP genes resulting in thermotolerance in plants (Queitsch et al., 2000). In further support for the role of *AtCBK3* in heat stress adaptation, Liu et al. (2008) observed that *cbk3* mutants of *Arabidopsis* exhibited reduced thermotolerance and overexpression of *AtCBK3* rescued the mutant plants (Table 1). Another likely mechanism by which CaM regulates heat shock response in plants is through regulation of protein phosphatases. The protein phosphatase AtPP7 in *Arabidopsis* exhibits Ca^2+^-dependent binding to CaM, and mutation in *AtPP7* resulted in decreased thermotolerance (Kutuzov et al., 2001; Liu et al., 2007; Table 1). Compared with cytosolic localization of AtCBK3 protein (Liu et al., 2008), the nuclear localization of AtPP7 (Liu et al., 2007) suggests that the latter might be activating the HSFs through its phosphatase activity as HSFs are reported to show enhanced transcriptional activity after dephosphorylation at some sites (Høj and Jakobsen, 1994).

Heat shock response in plants also appears to be regulated by CaM through interaction with HSPs such as HSP70 (Sun et al., 2000) and HSP90 (Virdi et al., 2009, 2011). The HSP90 (Zou et al., 1998) and HSP70 (Sun et al., 2000) have been proposed to act as repressors of HSFs in the absence of heat stress. It has been proposed that denatured proteins, produced as a result of unfolded protein response under heat stress, bind to HSP70/HSP90, thereby releasing the HSF that undergoes trimerization, phosphorylation, and nuclear localization leading to the transcription of genes encoding HSPs (Reindl et al., 1997; Yamada and Nishimura, 2008). Studies in our lab demonstrated that sorghum HSP90, SbHSP90, binds to CaM in a Ca^2+^-dependent manner (Virdi et al., 2009). It was also observed that the steady state levels of SbHSP90 are regulated through Ca^2+^/CaM pathway since the heat stress-induced increase in HSP90 levels was abrogated in the presence of exogenous Ca^2+^-channel blockers and CaM-antagonists (Virdi et al., 2011). On the basis of these observations, we proposed that in addition to denatured proteins, as suggested by Yamada and Nishimura (2008), dissociation of HSP90-HSF complex might also involve interaction of Ca^2+^-CaM with HSP90, thereby releasing the HSF to activate the transcription of HSP genes (Virdi et al., 2011). The role of HSP90 as a natural inhibitor of HSF is also supported by studies in *Arabidopsis* (Yamada et al., 2007) and sorghum (Virdi et al., 2011) where expression of *HSP* genes was shown to be enhanced by exogenous application of a specific inhibitor of HSP90, geldanamycin, even in the absence of heat shock. Further evidence for validation of the proposed model was also provided by recent studies that reported enhanced expression of *AtCBK3, AtPP7, AtHSF*, and *AtHSP* genes in absence of heat stress in the transgenic *Arabidopsis* that overexpressed *OsCaM1-1* (Wu et al., 2012). On the contrary, overexpression of *AtHSP90.3* lowered the expression of *AtHsfA1d, AtHsfA7a, AtHsfB1, AtHsp101*, and *AtHsp17*, leading to impaired thermotolerance of transgenic *Arabidopsis* (Xu et al., 2010). Therefore, regulation of heat shock response in plants by CaM through multiple pathways may ensure redundancy and enable them to cope up with thermal stress (Figure 2).

In addition to HSPs, plants are also reported to express heat-specific isoforms of FK506-binding proteins, FKBPs, which show peptidyl prolyl *cis-trans* isomerase activity (Schreiber, 1991). In plants, the multi-domain FKBPs, such as FKBP62, FKBP73, and FKBP77, are characterized by the presence of FKBP domains at N-termini, and CaM-binding and tetratricopeptide repeat domains at C-termini. Though wFKBP73 and wFKBP77 in wheat have been implicated in assembly of functional glucocorticoid complex with p23 and HSP90 (Owens-Grillo et al., 1996; Pratt and Toft, 1997), the implication of CaM in regulation of these proteins is still a matter of speculation and awaits validation. One of the FKBPs in *Arabidopsis*, AtFKBP62 or ROF1, is heat-inducible and forms a complex with HSP90, which is then translocated from cytosol to nucleus following exposure to thermal stress (Meiri and Breiman, 2009). The heat shock-induced localization of ROF1-HSP90 in the nucleus takes place as a result of interaction of transcription factor HSFA2 with HSP90 in this complex, leading to synthesis of sHSPs and development of thermotolerance. The role of CaM, if any, in the AtFKBP62-mediated stabilization of HSFA2 and its localization to nucleus, is not understood yet and needs further investigations.

## Concluding remarks and future directions

Modulation of intracellular Ca^2+^ levels in response to unfavorable conditions is one of the important components of signaling pathways that allow plants to adapt to changing environmental conditions. Of the various Ca^2+^ sensors, CaM is one of the most well-characterized proteins that decodes Ca^2+^ signals and regulates activities of diverse proteins. Approximately, 3% of the proteome in *Arabidopsis* has been reported to be involved in Ca^2+^ signaling (Reddy et al., 2011). In recent years, considerable progress has been made in our understanding of the role of CaM in regulating different cellular processes in plants. These studies have resulted in the identification and characterization of various proteins that are regulated by CaM. CaM not only acts as a sensor of Ca^2+^ but also regulates its intracellular levels by modulating the activity of Ca^2+^-ATPases. Detailed investigations into the specificity of different CaM isoforms for different Ca^2+^ pumps may reveal the role of these interactions in generating stimulus-specific signals. In addition to Ca^2+^ transport, CaM also affects uptake of heavy metal ions through modulation of channel proteins such as NtCBP4. Engineering of these proteins offers a promising alternative for developing transgenic plants that are able to tolerate toxic levels of heavy metal ions. Further, how CaM regulates activities of apyrases and Gly proteins, that confer tolerance to xenobiotic compounds, is still a matter of speculation. Understanding the physiological implications of interaction of CaM with apyrases and Gly enzymes may unravel strategies for enhancing stress tolerance in crops by transgenic technologies (Table 1).

Production of ROS under stress conditions is one of the important factors that cause damage to cellular components. Relieving autoinhibition of catalase by Ca^2+^-CaM, and regulation of NADPH oxidase and NAD kinase through Ca^2+^/CaM signaling pathway are indicative of complex feedback mechanisms that may allow the cells to maintain H_2_O_2_ homeostasis. Since ROS are also involved in biotic stress response, studies are required to determine the role of Ca^+2^-CaM in regulating cross-talk between biotic- and abiotic stress pathways that enable the cells to maintain appropriate levels of ROS. Regulation of TFs by Ca^+2^-CaM appears to be a crucial factor in stress adaptation response of plants. CaM-regulated TFs are involved in both activation and suppression of stress responses. For instance, CAMTA3 acts as a suppressor of plant immunity, whereas GTL1 functions as a negative regulator of drought tolerance (Table 1). Furthermore, a given TF can also affect two processes differently. For example, CAMTA3 acts as a negative regulator of immune response as well as a positive regulator of the cold stress response (Table 1). It appears that the stress-specific functions of a TF are dependent upon the effector proteins that bind to the promoter regions of these genes. Identification of upstream DNA elements and regulatory proteins that bind to these regions will provide further insights into the molecular mechanisms which determine multiple functionality of Ca^2+^-CaM-regulated TFs. Studies are also needed to understand the significance of different domains that facilitate interaction of TFs, such as NAC, with CaM in Ca^2+^-dependent and Ca^2+^-independent manner. This will provide insights into the mechanisms that regulate the interaction of CaM with these proteins, and may also allow engineering of specific domains for improving stress tolerance of crop plants. As of now, very few stress-regulated CaM-binding TFs have been identified but a growing body of data suggests that many more such proteins could exist (Table 1). Therefore, there is a need to carry out systematic analysis for identification and characterization of CaM-regulated TFs. Cross-talk between different regulatory pathways also needs to be investigated for developing a comprehensive view of stress response in plants.

Regulation of several processes such as maintenance of Ca^2+^ and H_2_O_2_ homeostasis, and heat shock response by CaM appears to be mediated through complex feedback mechanisms involving interaction of several different pathways. It is evident that CaM is acting as a hub for integration of different signal transduction pathways that enable cells to respond to different stimuli and maintain homeostasis. System biology approach would, therefore, be helpful in developing a deeper understanding of these mechanisms. Another aspect that needs to be explored further is to understand the cross-talk between regulation of different stress-modulated CaMBPs and phytohormones. With advances in protein microarray, transcriptome, and genome sequencing technologies, the number of stress-regulated CaMBPs that are identified in economically important crops is expected to increase further. Analysis of these proteins by using functional genomics approaches will, therefore, be an important factor in unraveling novel candidate genes for improving stress tolerance of crops by biotechnological interventions.

### Conflict of interest statement

The authors declare that the research was conducted in the absence of any commercial or financial relationships that could be construed as a potential conflict of interest.

## Abbreviations

ABA: abscisic acid
Ca^2+^: calcium
CaM: calmodulin
CaM-KMT: CaM N-methyletransferase
cAMP: cyclic adenosine monophosphate
cGMP: cyclic guanosine monophosphate
HS: heat shock
IAA: indole-3-acetic acid
IP3: inositol 1,4,5-trisphosphate
IP6: myo-inositol hexaphosphate
NLSs: nuclear-localization signals
PA: phosphatidic acid
TFs: transcription factors
ROS: reactive oxygen species.
