# Supplementary material for: Abiotic stress responses in plants: roles of calmodulin-regulated proteins
Source: Front Plant Sci. 2015 Oct 14;6:809. doi: 10.3389/fpls.2015.00809 (PMC4604306; doi:10.3389/fpls.2015.00809)
Supplement: Supplementary file 1 [file Table1.DOC]

|  | **Gene** | **Protein** | **Chr. no.** | **Locus id** | **Protein**  **accession no.** | **AA**  **residues** | **Types of**  **CaMBD** | **CaMBD**  **Score** | **CaMBD position**  **(AA)** |
| --- | --- | --- | --- | --- | --- | --- | --- | --- | --- |
| ***Arabidopsis thaliana*** | *AtGLYI1* | AtGLYI-1 | 1 | AT1G07645.1 | Q9LQP1 | 137 |  | 4 | 15-39; 115-134 |
|  | *AtGLYI2* | AtGLYI-2.1 | 1 | AT1G08110.1 | Q8H0V3 | 185 |  | 0 | 0 |
|  |  | AtGLYI-2.2 |  | AT1G08110.2 | Q8H0V3 | 185 |  | 0 | 0 |
|  |  | AtGLYI-2.3 |  | AT1G08110.3 | Q8H0V3 | 185 |  | 0 | 0 |
|  |  | AtGLYI-2.4 |  | AT1G08110.4 | B9DH52 | 235 |  | 0 | 0 |
|  | *AtGLYI3* | AtGLYI-3.1 | 1 | AT1G11840.1 | O65398 | 235 |  | 2 | 97-118;159-180 |
|  |  | AtGLYI-3.2 |  | AT1G11840.2 | B9DGT0 | 283 |  | 2 | 97-118;159-180 |
|  |  | AtGLYI-3.3 |  | AT1G11840.3 | F4IAH9 | 283 |  | 2 | 135-157;159-229 |
|  |  | AtGLYI-3.4 |  | AT1G11840.4 | Q940A4 | 283 |  | 2 | 96-118;159-180 |
|  |  | AtGLYI-3.5 |  | AT1G11840.5 | B9DFN6 | 232 | unclassified  CaMBD at 221 | 9 | 221-230 |
|  | *AtGLYI4* | AtGLYI-4.1 | 1 | AT1G15380.1 | Q9XI31 | 186 |  | 0 | 0 |
|  |  | AtGLYI-4.2 |  | AT1G15380.2 | Q56Z37 | 60 |  | 0 | 0 |
|  | *AtGLYI5* | AtGLYI-5 | 1 | AT1G64185.1 | Q8LGF9 | 118 |  | 3 | 18-39 |
|  | *AtGLYI6* | AtGLYI-6.1 | 1 | AT1G67280.1 | Q8W593 | 350 |  | 3 | 170-189;298-319 |
|  |  | AtGLYI-6.2 |  | AT1G67280.2 | Q8W593-2 | 262 |  | 3 | 81-101;209-231 |
|  | *AtGLYI7* | AtGLYI-7.1 | 1 | AT1G80160.1 | Q8LD97 | 167 |  | 0 | 0 |
|  |  | AtGLYI-7.2 |  | AT1G80160.2 | A8MS67 | 141 |  | 0 | 0 |
|  | *AtGLYI8* | AtGLYI-8 | 2 | AT2G28420.1 | Q9SKM8 | 184 |  | 0 | 0 |
|  | *AtGLYI9* | AtGLYI-9.1 | 2 | AT2G32090.1 | Q9SKZ0 | 135 |  | 0 | 0 |
|  |  | AtGLYI-9.2 | 2 | AT2G32090.2 | F4ISS2 | 113 |  | 0 | 0 |
|  | *AtGLYI10* | AtGLYI-10 | 5 | AT5G41650.1 | Q9FFR5 | 117 |  | 0 | 0 |
|  | *AtGLYI11* | AtGLYI-11 | 5 | AT5G57040.1 | Q9LTR8 | 197 |  | 9 | 29-60 |
|  | *AtGLYII1* | AtGLYI-I1.1 | 1 | AT1G06130.1 | Q8LDW8 | 331 |  | 2 | 2-22;263-282 |
|  |  | AtGLYI-I1.2 |  | AT1G06130.2 | Q8LDW8 | 330 |  | 2 | 2-22; 263-282 |
|  | *AtGLYII2* | AtGLYI-I2.1 | 1 | AT1G53580.1 | Q9C8L4 | 294 |  | 0 | 0 |
|  |  | AtGLYI-I2.2 |  | AT1G53580.2 | F4HRK0 | 294 |  | 0 | 0 |
|  | *AtGLYII3* | AtGLYI-I3.1 | 2 | AT2G31350.1 | Q9SID3 | 324 |  | 1 | 129-150;258-287 |
|  |  | AtGLYI-I3.2 |  | AT2G31350.2 | Q9SID3-2 | 323 |  | 1 | 129-148;257-276 |
|  | *AtGLYII4* | AtGLYI-I4.1 | 2 | AT2G43430.1 | O24495 | 331 |  | 3 | 49-70 |
|  |  | AtGLYI-I4.2 |  | AT2G43430.2 | F4IR49 | 313 |  | 3 | 31-54 |
|  | *AtGLYII5* | AtGLYI-I5 | 3 | AT3G10850.1 | O24496 | 258 |  | 3 | 33-54 |
| ***Oryza sativa*** | *OsGLYI1* | OsGLYI-1 | 1 | LOC_Os01g07850.1 |  | 215 | IQ motif at 98 | 3 | 33-54;98-128 |
|  | *OsGLYI2* | OsGLYI-2 | 2 | LOC_Os02g17920.1 |  | 377 |  | 5 | 47-70;105-124;194-216 |
|  | *OsGLYI3* | OsGLYI-3 | 3 | LOC_Os03g16940.1 |  | 216 |  | 7 | 97-134;189-209 |
|  | *OsGLYI4* | OsGLYI-4 | 3 | LOC_Os03g45720.1 |  | 141 |  | 0 | 0 |
|  | *OsGLYI5* | OsGLYI-5 | 4 | LOC_Os04g45590.1 |  | 175 |  | 2 | 45-65 |
|  | *OsGLYI6* | OsGLYI-6.1 | 5 | LOC_Os05g07940.1 |  | 208 |  | 0 | 0 |
|  |  | OsGLYI-6.2 |  | LOC_Os05g07940.2 |  | 176 |  | 1 | 18-37 |
|  |  | OsGLYI-6.3 |  | LOC_Os05g07940.3 |  | 106 |  | 0 | 0 |
|  |  | OsGLYI-6.4 |  | LOC_Os05g07940.4 |  | 123 |  | 6 | 57-81 |
|  |  | OsGLYI-6.5 |  | LOC_Os05g07940.5 |  | 136 |  | 0 | 0 |
|  | *OsGLYI7* | OsGLYI-7.1 | 5 | LOC_Os05g14194.1 |  | 350 |  | 0 | 0 |
|  |  | OsGLYI-7.2 |  | LOC_Os05g14194.2 |  | 274 |  | 0 | 0 |
|  | *OsGLYI8* | OsGLYI-8 | 5 | LOC_Os05g22970.1 |  | 237 |  | 13 | 17-47 |
|  | *OsGLYI9* | OsGLYI-9.1 | 7 | LOC_Os07g06660.1 |  | 188 |  | 6 | 79-100;150-174 |
|  |  | OsGLYI-9.2 |  | LOC_Os07g06660.2 |  | 178 |  | 3 | 79-100 |
|  | *OsGLYI10* | OsGLYI-10 | 7 | LOC_Os07g46360.1 |  | 219 |  | 0 | 0 |
|  | *OsGLYI11* | OsGLYI-11.1 | 8 | LOC_Os08g09250.1 |  | 292 |  | 2 | 168-188 |
|  |  | OsGLYI-11.2 |  | LOC_Os08g09250.2 |  | 291 |  | 2 | 168-188 |
|  |  | OsGLYI-11.3 |  | LOC_Os08g09250.3 |  | 181 |  | 0 | 0 |
|  | *OsGLYII1* | OsGLYII-1.1 | 1 | LOC_Os01g47690.1 |  | 305 |  | 5 | 47-80; 135-157 |
|  |  | OsGLYII-1.2 |  | LOC_Os01g47690.2 |  | 280 |  | 5 | 47-80; 135-157 |
|  | *OsGLYII2* | OsGLYII-2 | 3 | LOC_Os03g21460.1 |  | 258 |  | 0 | 0 |
|  | *OsGLYII3* | OsGLYII-3 | 9 | LOC_Os09g34100.1 |  | 336 |  | 4 | 144-169 |

**Table S1:** Analysis of calmodulin-binding domains of glyoxalase I (GlyI) and glyoxalase II (GlyII) proteins of Arabidopsis and rice.

Chr. no.: chromosome number; AA: amino acid residues; CaMBD: calmodulin-binding domain.
