# Supplementary material for: Abiotic stress responses in plants: roles of calmodulin-regulated proteins
Source: Front Plant Sci. 2015 Oct 14;6:809. doi: 10.3389/fpls.2015.00809 (PMC4604306; doi:10.3389/fpls.2015.00809)
Supplement: Supplementary file 2 [file Table2.DOC]

|  | **1** | **2** | **3** | **4** | **5** | **6** | **7** | **8** | **9** | **10** | **11** | **12** | **13** | **14** | **15** |
| --- | --- | --- | --- | --- | --- | --- | --- | --- | --- | --- | --- | --- | --- | --- | --- |
| **1. GhCAT3.1** |  | 83.8 | 83.8 | 78.4 | 78.4 | 78.4 | 75.7 | 78.4 | 73.0 | 75.7 | 78.4 | 78.4 | 73.0 | 75.7 | 75.7 |
| **2. GhCAT3.2** | 94.6 |  | 75.7 | 75.7 | 78.4 | 73.0 | 67.6 | 78.4 | 73.0 | 73.0 | 78.4 | 78.4 | 64.9 | 73.0 | 73.0 |
| **3. FaCAT3** | 89.2 | 86.5 |  | 81.1 | 75.7 | 78.4 | 70.3 | 75.7 | 75.7 | 78.4 | 75.7 | 75.7 | 70.3 | 70.3 | 78.4 |
| **4. BdCAT3.1** | 83.8 | 81.1 | 89.2 |  | 78.4 | 78.4 | 73.0 | 78.4 | 94.6 | 97.3 | 78.4 | 78.4 | 73.0 | 78.4 | 97.3 |
| **5. BdCAT3.2** | 83.8 | 83.8 | 83.8 | 86.5 |  | 70.3 | 64.9 | 100.0 | 73.0 | 75.7 | 100.0 | 100.0 | 64.9 | 86.5 | 75.7 |
| **6. LjCAT3** | 89.2 | 86.5 | 89.2 | 86.5 | 81.1 |  | 78.4 | 70.3 | 75.7 | 78.4 | 70.3 | 70.3 | 75.7 | 70.3 | 75.7 |
| **7. MtCAT3** | 83.8 | 83.8 | 83.8 | 83.8 | 75.7 | 89.2 |  | 64.9 | 75.7 | 75.7 | 64.9 | 64.9 | 73.0 | 70.3 | 70.3 |
| **8. TaCAT3.1** | 83.8 | 83.8 | 83.8 | 86.5 | 100.0 | 81.1 | 75.7 |  | 73.0 | 75.7 | 100.0 | 100.0 | 64.9 | 86.5 | 75.7 |
| **9. TaCAT3.2** | 83.8 | 81.1 | 89.2 | 100.0 | 86.5 | 86.5 | 83.8 | 86.5 |  | 97.3 | 73.0 | 73.0 | 73.0 | 75.7 | 91.9 |
| **10. TuCAT3.1** | 83.8 | 81.1 | 89.2 | 100.0 | 86.5 | 86.5 | 83.8 | 86.5 | 100.0 |  | 75.7 | 75.7 | 73.0 | 75.7 | 94.6 |
| **11. TuCAT3.2** | 83.8 | 83.8 | 83.8 | 86.5 | 100.0 | 81.1 | 75.7 | 100.0 | 86.5 | 86.5 |  | 100.0 | 64.9 | 86.5 | 75.7 |
| **12. AetCAT3** | 83.8 | 83.8 | 83.8 | 86.5 | 100.0 | 81.1 | 75.7 | 100.0 | 86.5 | 86.5 | 100.0 |  | 64.9 | 86.5 | 75.7 |
| **13. AtCAT3** | 86.5 | 83.8 | 91.9 | 89.2 | 78.4 | 89.2 | 83.8 | 78.4 | 86.5 | 89.2 | 78.4 | 78.4 |  | 73.0 | 70.3 |
| **14. OsCATC** | 83.8 | 83.8 | 86.5 | 89.2 | 91.9 | 83.8 | 81.1 | 91.9 | 89.2 | 89.2 | 91.9 | 91.9 | 81.1 |  | 75.7 |
| **15. SbCAT3** | 83.8 | 81.1 | 89.2 | 100.0 | 86.5 | 86.5 | 83.8 | 86.5 | 100.0 | 100.0 | 86.5 | 86.5 | 89.2 | 89.2 |  |

**Table S2:** Identity and similarity analysis of calmodulin (CaM)-binding domain of different plant catalases.

*Gossypium hirsutum* (GhCAT3.1: P17598, GhCAT3.2: P30567); *Fragaria ananassa* (FaCAT3: M9R036); *Brachypodium distachyon* (BdCAT3.1: I1GV73, BdCAT3.2: I1HA44); *Lotus japonicus* (LjCAT3: A0PG71); *Medicago truncatula* (MdCAT3: A0A072V3A8); *Triticum aestivum* (TaCAT3.1: Q43206, TaCAT3.2: P55313); *Triticum urartu* (TuCAT3.1: M8A9J7, TuCAT3.2: T1N7Q8); *Aegilops tauschii* (AetCAT3: M8ATK7); *Arabidopsis thaliana* (AtCAT3: AAC49807.1); *Oryza sativa* (NP_001048861.1); *Sorghum bicolor* (XP_002437631.1).
