# Supplementary material for: Abiotic stress responses in plants: roles of calmodulin-regulated proteins
Source: Front Plant Sci. 2015 Oct 14;6:809. doi: 10.3389/fpls.2015.00809 (PMC4604306; doi:10.3389/fpls.2015.00809)
Supplement: Supplementary file 3 [file Table3.DOC]

|  |  | **1** | **2** | **3** | **4** | **5** | **6** | **7** | **8** | **9** | **10** | **11** | **12** | **13** | **14** | **15** | **16** | **17** | **18** | **19** | **20** |
| --- | --- | --- | --- | --- | --- | --- | --- | --- | --- | --- | --- | --- | --- | --- | --- | --- | --- | --- | --- | --- | --- |
| ***Arabidopsis thaliana*** | **1. AtCAMTA1** | **Similarity**  **Identity** | 70 | 41.4 | 33.3 | 30.6 | 29.5 | 44.4 | 46.4 | 45.1 | 32.4 | 30.3 | 45.4 | 32.7 | 45.8 | 33.9 | 26.9 | 44 | 30.6 | 29.4 | 30 |
|  | **2. AtCAMTA2** | 82.5 |  | 42.3 | 34.3 | 31.9 | 28.8 | 43.5 | 46.5 | 46.4 | 31.3 | 30 | 45.4 | 31.6 | 46.7 | 34.9 | 26.4 | 46 | 30.7 | 30.9 | 30.4 |
|  | **3. AtCAMTA3** | 61.7 | 63.9 |  | 33.1 | 29.8 | 27.8 | 42.5 | 43.2 | 43.1 | 28.3 | 28.4 | 43.5 | 29.3 | 43 | 32.4 | 25.4 | 42.8 | 29 | 28.3 | 27.8 |
|  | **4. AtCAMTA4** | 55.7 | 56.6 | 53.7 |  | 30.8 | 29.4 | 32.8 | 31.7 | 33 | 36.4 | 29.5 | 33.1 | 35.6 | 33.8 | 40 | 21.6 | 33.4 | 22.1 | 30.1 | 29.7 |
|  | **5. AtCAMTA5** | 49.3 | 50 | 48.6 | 49.1 |  | 66.2 | 30.7 | 32.7 | 31.6 | 29.7 | 43.2 | 32.3 | 28.5 | 32.4 | 31.3 | 19.8 | 31.6 | 22.9 | 44.8 | 44.1 |
|  | **6. AtCAMTA6** | 46.3 | 46.8 | 47.3 | 46.2 | 78.1 |  | 29 | 30.4 | 28.6 | 29.9 | 43.1 | 30.3 | 31.6 | 28.9 | 29.5 | 22.2 | 29.2 | 22.6 | 42.6 | 42.4 |
| ***Sorghum bicolor*** | **7. XP_002467764.1** | 62 | 62.9 | 62.6 | 55.1 | 50.8 | 47.3 |  | 50.9 | 51.7 | 31.5 | 29.7 | 68.7 | 31.9 | 49.6 | 33.7 | 43.2 | 51 | 48.1 | 30.7 | 29.9 |
|  | **8. XP_002465719.1** | 65 | 65.1 | 62.2 | 54 | 52.2 | 48.3 | 68.1 |  | 71.5 | 32 | 30.7 | 54.2 | 32.9 | 76.3 | 34.5 | 32.1 | 71.9 | 35.2 | 31.3 | 31.3 |
|  | **9. XP_002489212.1** | 61.9 | 64.6 | 62.6 | 54 | 51.3 | 47.4 | 69.3 | 82.4 |  | 30.2 | 30.6 | 53.4 | 32.8 | 73.3 | 33.4 | 30.5 | 80.5 | 35.4 | 29.8 | 29.3 |
|  | **10. XP_002456865.1** | 51.3 | 50.1 | 46 | 52.6 | 49.4 | 50.9 | 48.1 | 49.5 | 48.2 |  | 28.7 | 31.3 | 65.8 | 30 | 38.8 | 22.3 | 30.4 | 22.5 | 30.2 | 30.1 |
|  | **11. XP_002462876.1** | 51 | 50.7 | 48.8 | 48.7 | 64.4 | 61.4 | 49.5 | 52.1 | 52 | 49.3 |  | 29.2 | 29.7 | 31.1 | 30.7 | 19.7 | 30.3 | 22.2 | 70.9 | 70 |
|  | **12. XP_002463205.1** | 64.5 | 64.2 | 63 | 55 | 51.9 | 48.7 | 80.1 | 70.4 | 69.4 | 49 | 51.3 |  | 32.8 | 51.8 | 35.2 | 39.5 | 53 | 52.5 | 31.1 | 30.8 |
| ***Oryza sativa*** | **13. LOC_Os01g69910.1** | 52.4 | 50.5 | 48 | 53.1 | 50.1 | 52.3 | 49.5 | 52 | 50 | 78.8 | 49.4 | 51 |  | 31.7 | 38.2 | 22.7 | 32.4 | 22.2 | 30.7 | 30.5 |
|  | **14. LOC_Os03g09100.1** | 63 | 64.4 | 62.8 | 54.3 | 51.7 | 47.4 | 68.6 | 85.1 | 84.9 | 47.3 | 51 | 69.8 | 49.9 |  | 34.7 | 29.7 | 73.1 | 34 | 30.2 | 30 |
|  | **15. LOC_Os04g31900.1** | 55.8 | 54.6 | 52 | 60.1 | 50.7 | 47.4 | 53.9 | 52.8 | 53.3 | 56.1 | 50.3 | 55.2 | 55.5 | 53.9 |  | 21.2 | 33.5 | 24.4 | 30.2 | 30.7 |
|  | **16. LOC_Os03g27080.1** | 37.4 | 37 | 36.7 | 33.4 | 33.9 | 38.1 | 47 | 40.8 | 40.1 | 35.4 | 32.7 | 45.5 | 35 | 39.7 | 31.6 |  | 30.2 | 17.7 | 20.1 | 20 |
|  | **17. LOC_Os10g22950.1** | 62.4 | 63.2 | 63.2 | 54.7 | 52 | 48 | 69.2 | 82.9 | 89.9 | 46.9 | 50.1 | 70 | 50.3 | 85.2 | 52.7 | 39.8 |  | 35.9 | 30.1 | 29.7 |
|  | **18. LOC_Os07g43030.1** | 43 | 43.1 | 42.4 | 38.1 | 38.6 | 40.1 | 55.1 | 46.4 | 46.6 | 40.8 | 39 | 59.5 | 40.5 | 46.2 | 38.3 | 31.3 | 47.2 |  | 21.6 | 21.6 |
|  | **19. LOC_Os07g30774.1** | 50 | 50.9 | 48.6 | 49.1 | 65.4 | 60.9 | 50.7 | 52.3 | 51 | 50.1 | 83.5 | 51.1 | 49.9 | 49.9 | 50.5 | 33.3 | 49.7 | 38.4 |  | 98.7 |
|  | **20. OsCBT** | 50.8 | 50.5 | 48 | 48.5 | 64.4 | 60.4 | 49.9 | 51.3 | 50.6 | 49.2 | 82.8 | 50.6 | 49.9 | 49.6 | 51.1 | 33.2 | 49.6 | 38.5 | 99.1 |  |

**Table S3:** Identity and similarity analysis of different calmodulin (CaM)-binding transcription activator (CAMTA) proteins of Arabidopsis, rice and sorghum.

**Similarity**

**Identity**
