# Supplementary material for: Abiotic stress responses in plants: roles of calmodulin-regulated proteins
Source: Front Plant Sci. 2015 Oct 14;6:809. doi: 10.3389/fpls.2015.00809 (PMC4604306; doi:10.3389/fpls.2015.00809)
Supplement: Supplementary file 4 [file Table4.DOC]

|  | **Gene** | **Protein** | **Locus id/protein accession no.** | **AA**  **residues** | **Types of CaMBD** | **CaMBD Score** | **CAMBD1**  **(AA)** | **CAMBD2**  **(AA)** |
| --- | --- | --- | --- | --- | --- | --- | --- | --- |
| ***Arabidopsis thaliana*** | *AtCAMTA1* | AtCAMTA1 | NP_001119195.1 | 1007 | unclassified CAMBD at 868, 872 | 9 |  | 871-890 |
|  | *AtCAMTA2* | AtCAMTA2 | BAF01265.1 | 1050 | unclassified CAMBD at 917 | 9 |  | 881-900 |
|  | *AtCAMTA3* | AtCAMTA3 | AEC07290.1 | 1032 | 899 | 9 |  | 912-931 |
|  | *AtCAMTA4* | AtCAMTA4 | AEE34626.1 | 1016 | IQ motif at aa 801, 858, 881 | 20 |  | 905-924 |
|  | *AtCAMTA5* | AtCAMTA5 | AEE83703.1 | 923 | IQ motif at aa 726, 760, 802 | 20 | 764-770 | 826-844 |
|  | *AtCAMTA6* | AtCAMTA6 | BAD95048.1 | 153 | IQ motif at 41 | 20 | 678-684 | 740-760 |
| ***Oryza sativa*** | *OsCAMTA1* | OsCAMTA1 | LOC_Os01g69910.1 | 878 | IQ motif at 672, 717 | 17 |  | 763-783 |
|  | *OsCAMTA2* | OsCAMTA2 | LOC_Os03g09100.1 | 1029 | unclassified CAMBD at 894 | 9 |  | 896-916 |
|  | *OsCAMTA3* | OsCAMTA3 | LOC_Os04g31900.1 | 1003 | IQ motif at 805, 863, 886 | 18 |  | 909-929 |
|  | *OsCAMTA4* | OsCAMTA4 | LOC_Os03g27080.1 | 545 | IQ motif at 372; 395; 459 | 20 |  | 418-438 |
|  | *OsCAMTA5* | OsCAMTA5 | LOC_Os10g22950.1 | 1023 | IQ motif at 844;867;938 | 20 |  | 890-910 |
|  | *OsCAMTA6* | OsCAMTA6 | LOC_Os07g43030.1 | 720 |  | 18 |  | 00-00 |
|  | *OsCAMTA7* | OsCAMTA7 | LOC_Os07g30774.1 | 927 | IQ motif at 726;760; 779; 802 | 20 | 764-770 | 825-845 |
|  | *OsCBT* | OsCBT | AAQ07306.1 | 927 | IQ motif at 726; 760; 779; 802 | 20 | 764-770 | 825-845 |
| ***Sorghum bicolor*** | *SbCAMTA1* | SbCAMTA1 | XP_002467764.1 | 1024 | IQ motif at 87; 851 | 20 |  | 897-917 |
|  | *SbCAMTA2* | SbCAMTA2 | XP_002465719.1 | 994 | unclassified CAMBD at 860 | 9 |  | 862-882 |
|  | *SbCAMTA3* | SbCAMTA3 | XP_002489212.1 | 1021 | unclassified CAMBD 889 | 9 |  | 892-911 |
|  | *SbCAMTA4* | SbCAMTA4 | XP_002456865.1 | 845 | IQ motif at 11; 668; 715; 738 | 17 |  | 761-781 |
|  | *SbCAMTA5* | SbCAMTA5 | XP_002462876.1 | 946 | IQ motif at 755; 789; 808; 831 | 20 | 793-799 | 854-874 |
|  | *SbCAMTA6* | SbCAMTA6 | XP_002463205.1 | 1012 | IQ motif at 835; 858 | 20 |  | 881-901 |

**Table S4:** *In silico* analysis for identification of putative calmodulin (CaM)-binding domains of CaM-binding transcription activator (CAMTA) proteins of Arabidopsis, rice and sorghum.

Chr. no.: chromosome number; AA: amino acid residues; CaMBD: calmodulin-binding domain.
