# Supplementary material for: Abiotic stress responses in plants: roles of calmodulin-regulated proteins
Source: Front Plant Sci. 2015 Oct 14;6:809. doi: 10.3389/fpls.2015.00809 (PMC4604306; doi:10.3389/fpls.2015.00809)
Supplement: Supplementary file 5 [file Table5.DOC]

| **CaMBD1** | | | | | | | | | | | | | | | | | | | | |
| --- | --- | --- | --- | --- | --- | --- | --- | --- | --- | --- | --- | --- | --- | --- | --- | --- | --- | --- | --- | --- |
|  |  | **1** | **2** | **3** | **4** | **5** | **6** | **7** | **8** | **9** | **10** | **11** | **12** | **13** | **14** | **15** | **16** | **17** | **18** | **19** |
| ***Arabidopsis thaliana*** | **1. AtCAMTA1**  **Similarity**  **Identity** |  | 18.2 | 23.1 | 18.2 | 0 | 7.1 | 45.5 | 36.4 | 36.4 | 18.2 | 0 | 45.5 | 18.2 | 36.4 | 17.6 | 36.4 | 36.4 | 0 | 0 |
|  | **2. AtCAMTA2** | 45.5 |  | 46.2 | 9.1 | 7.1 | 0 | 27.3 | 18.2 | 36.4 | 18.2 | 0 | 45.5 | 18.2 | 18.2 | 14.3 | 27.3 | 36.4 | 0 | 0 |
|  | **3. AtCAMTA3** | 38.5 | 61.5 |  | 7.7 | 7.1 | 7.1 | 30.8 | 15.4 | 30.8 | 7.7 | 7.1 | 30.8 | 7.7 | 15.4 | 0 | 30.8 | 30.8 | 7.1 | 7.1 |
|  | **4. AtCAMTA4** | 45.5 | 18.2 | 30.8 |  | 13.3 | 21.4 | 9.1 | 0 | 10 | 20 | 26.7 | 20 | 10 | 0 | 14.3 | 9.1 | 10 | 26.7 | 26.7 |
|  | **5. AtCAMTA5** | 14.3 | 21.4 | 21.4 | 28.6 |  | 64.3 | 0 | 0 | 0 | 7.1 | 50 | 0 | 7.1 | 7.1 | 12.5 | 7.1 | 0 | 50 | 50 |
|  | **6. AtCAMTA6** | 14.3 | 14.3 | 28.6 | 28.6 | 85.7 |  | 14.3 | 7.1 | 7.1 | 0 | 42.9 | 7.1 | 0 | 14.3 | 0 | 21.4 | 7.1 | 42.9 | 42.9 |
| ***Sorghum bicolor*** | **7. XP_002467764.1** | 45.5 | 45.5 | 53.8 | 45.5 | 28.6 | 42.9 |  | 54.5 | 54.5 | 18.2 | 0 | 54.5 | 18.2 | 54.5 | 17.6 | 81.8 | 54.5 | 0 | 0 |
|  | **8. XP_002465719.1** | 36.4 | 36.4 | 38.5 | 20 | 21.4 | 21.4 | 54.5 |  | 70 | 10 | 0 | 50 | 20 | 90 | 21.4 | 45.5 | 80 | 0 | 0 |
|  | **9. XP_002489212.1** | 36.4 | 54.5 | 53.8 | 30 | 21.4 | 28.6 | 54.5 | 70 |  | 20 | 0 | 60 | 20 | 70 | 14.3 | 54.5 | 90 | 0 | 0 |
|  | **10. XP_002456865.1** | 27.3 | 27.3 | 23.1 | 30 | 14.3 | 21.4 | 27.3 | 20 | 30 |  | 7.1 | 33.3 | 85.7 | 10 | 14.3 | 18.2 | 20 | 7.1 | 7.1 |
|  | **11. XP_002462876.1** | 21.4 | 28.6 | 42.9 | 35.7 | 78.6 | 78.6 | 35.7 | 14.3 | 14.3 | 21.4 |  | 0 | 7.1 | 7.1 | 7.1 | 0 | 0 | 92.9 | 92.9 |
|  | **12. XP_002463205.1** | 54.5 | 54.5 | 46.2 | 40 | 21.4 | 14.3 | 63.6 | 60 | 70 | 33.3 | 21.4 |  | 33.3 | 50 | 7.1 | 45.5 | 50 | 0 | 0 |
| ***Oryza sativa*** | **13. LOC_Os01g69910.1** | 27.3 | 27.3 | 23.1 | 20 | 7.1 | 14.3 | 18.2 | 30 | 30 | 85.7 | 14.3 | 33.3 |  | 20 | 14.3 | 18.2 | 30 | 7.1 | 7.1 |
|  | **14. LOC_Os03g09100.1** | 36.4 | 36.4 | 38.5 | 20 | 28.6 | 28.6 | 54.5 | 90 | 70 | 20 | 21.4 | 60 | 20 |  | 21.4 | 45.5 | 80 | 7.1 | 7.1 |
|  | **15. LOC_Os04g31900.1** | 21.4 | 35.7 | 14.3 | 35.7 | 28.6 | 14.3 | 28.6 | 21.4 | 14.3 | 35.7 | 21.4 | 7.1 | 35.7 | 21.4 |  | 11.8 | 21.4 | 7.1 | 7.1 |
|  | **16. LOC_Os03g27080.1** | 36.4 | 45.5 | 53.8 | 36.4 | 35.7 | 50 | 81.8 | 45.5 | 54.5 | 36.4 | 42.9 | 54.5 | 27.3 | 45.5 | 21.4 |  | 54.5 | 0 | 0 |
|  | **17. LOC_Os10g22950.1** | 36.4 | 54.5 | 53.8 | 30 | 21.4 | 28.6 | 54.5 | 80 | 90 | 30 | 21.4 | 60 | 40 | 80 | 21.4 | 54.5 |  | 0 | 0 |
|  | **18. LOC_Os07g30774.1** | 14.3 | 21.4 | 35.7 | 35.7 | 78.6 | 78.6 | 28.6 | 7.1 | 7.1 | 14.3 | 92.9 | 21.4 | 7.1 | 14.3 | 21.4 | 35.7 | 7.1 |  | 100 |
|  | **19. OsCBT** | 14.3 | 21.4 | 35.7 | 35.7 | 78.6 | 78.6 | 28.6 | 7.1 | 7.1 | 14.3 | 92.9 | 21.4 | 7.1 | 14.3 | 21.4 | 35.7 | 7.1 | 100 |  |
| **CaMBD2** | | | | | | | | | | | | | | | | | | | | |
| ***Arabidopsis thaliana*** | **1. AtCAMTA1** |  | 95.0 | 85.0 | 70.0 | 70.0 | 65.0 | 70.0 | 80.0 | 75.0 | 70.0 | 70.0 | 75.0 | 75.0 | 75.0 | 80.0 | 80.0 | 70.0 | 70.0 | 65.0 |
|  | **2. AtCAMTA2** | 100.0 |  | 85.0 | 70.0 | 70.0 | 65.0 | 70.0 | 80.0 | 75.0 | 70.0 | 70.0 | 75.0 | 75.0 | 75.0 | 80.0 | 80.0 | 70.0 | 70.0 | 65.0 |
|  | **3. AtCAMTA3** | 95.0 | 100.0 |  | 75.0 | 75.0 | 70.0 | 75.0 | 90.0 | 85.0 | 80.0 | 75.0 | 80.0 | 80.0 | 85.0 | 90.0 | 85.0 | 80.0 | 70.0 | 65.0 |
|  | **4. AtCAMTA4** | 90.0 | 90.0 | 90.0 |  | 55.0 | 60.0 | 65.0 | 75.0 | 70.0 | 70.0 | 55.0 | 70.0 | 80.0 | 70.0 | 75.0 | 75.0 | 65.0 | 60.0 | 55.0 |
|  | **5. AtCAMTA5** | 80.0 | 80.0 | 80.0 | 75.0 |  | 90.0 | 65.0 | 65.0 | 60.0 | 60.0 | 85.0 | 70.0 | 60.0 | 60.0 | 70.0 | 65.0 | 65.0 | 80.0 | 75.0 |
|  | **6. AtCAMTA6** | 85.0 | 85.0 | 85.0 | 80.0 | 95.0 |  | 60.0 | 60.0 | 55.0 | 60.0 | 80.0 | 65.0 | 60.0 | 55.0 | 65.0 | 60.0 | 60.0 | 75.0 | 70.0 |
| ***Sorghum bicolor*** | **7. XP_002467764.1** | 85.0 | 85.0 | 85.0 | 80.0 | 80.0 | 85.0 |  | 85.0 | 80.0 | 60.0 | 75.0 | 90.0 | 70.0 | 80.0 | 70.0 | 85.0 | 85.0 | 80.0 | 75.0 |
|  | **8. XP_002465719.1** | 95.0 | 100.0 | 100.0 | 90.0 | 80.0 | 85.0 | 85.0 |  | 95.0 | 70.0 | 75.0 | 90.0 | 80.0 | 95.0 | 80.0 | 95.0 | 90.0 | 80.0 | 75.0 |
|  | **9. XP_002489212.1** | 95.0 | 100.0 | 100.0 | 90.0 | 80.0 | 85.0 | 85.0 | 100.0 |  | 65.0 | 70.0 | 85.0 | 75.0 | 100.0 | 75.0 | 90.0 | 95.0 | 75.0 | 70.0 |
|  | **10. XP_002456865.1** | 90.0 | 90.0 | 90.0 | 90.0 | 75.0 | 80.0 | 80.0 | 90.0 | 90.0 |  | 60.0 | 65.0 | 90.0 | 65.0 | 75.0 | 70.0 | 60.0 | 55.0 | 50.0 |
|  | **11. XP_002462876.1** | 85.0 | 85.0 | 85.0 | 80.0 | 95.0 | 100.0 | 85.0 | 85.0 | 85.0 | 80.0 |  | 80.0 | 60.0 | 70.0 | 70.0 | 75.0 | 75.0 | 95.0 | 90.0 |
|  | **12. XP_002463205.1** | 90.0 | 90.0 | 90.0 | 85.0 | 90.0 | 95.0 | 90.0 | 90.0 | 90.0 | 85.0 | 95.0 |  | 75.0 | 85.0 | 75.0 | 95.0 | 90.0 | 85.0 | 80.0 |
| ***Oryza sativa*** | **13. LOC_Os01g69910.1** | 90.0 | 90.0 | 90.0 | 90.0 | 75.0 | 80.0 | 80.0 | 90.0 | 90.0 | 100.0 | 80.0 | 85.0 |  | 75.0 | 75.0 | 80.0 | 70.0 | 65.0 | 60.0 |
|  | **14. LOC_Os03g09100.1** | 95.0 | 100.0 | 100.0 | 90.0 | 80.0 | 85.0 | 85.0 | 100.0 | 100.0 | 90.0 | 85.0 | 90.0 | 90.0 |  | 75.0 | 90.0 | 95.0 | 75.0 | 70.0 |
|  | **15. LOC_Os04g31900.1** | 95.0 | 90.0 | 90.0 | 90.0 | 75.0 | 80.0 | 80.0 | 90.0 | 90.0 | 85.0 | 80.0 | 85.0 | 85.0 | 90.0 |  | 80.0 | 70.0 | 65.0 | 60.0 |
|  | **16. LOC_Os03g27080.1** | 95.0 | 95.0 | 95.0 | 90.0 | 85.0 | 90.0 | 85.0 | 95.0 | 95.0 | 90.0 | 90.0 | 95.0 | 90.0 | 95.0 | 90.0 |  | 85.0 | 80.0 | 75.0 |
|  | **17. LOC_Os10g22950.1** | 90.0 | 95.0 | 95.0 | 85.0 | 85.0 | 90.0 | 90.0 | 95.0 | 95.0 | 85.0 | 90.0 | 95.0 | 85.0 | 95.0 | 85.0 | 90.0 |  | 80.0 | 75.0 |
|  | **18. LOC_Os07g30774.1** | 85.0 | 85.0 | 85.0 | 80.0 | 95.0 | 100.0 | 85.0 | 85.0 | 85.0 | 80.0 | 100.0 | 95.0 | 80.0 | 85.0 | 80.0 | 90.0 | 90.0 |  | 95.0 |
|  | **19. OsCBT** | 80.0 | 80.0 | 80.0 | 75.0 | 90.0 | 95.0 | 80.0 | 80.0 | 80.0 | 75.0 | 95.0 | 90.0 | 75.0 | 80.0 | 75.0 | 85.0 | 85.0 | 95.0 |  |

**Table S5:** Identity and similarity analysis of calmodulin (CaM)-binding domains 1 and 2 (CaMBD1 and CaMBD2) of different CaM-binding transcription activator (CAMTA) proteins of Arabidopsis, rice and sorghum.

**Similarity**

**Identity**
