# Supplementary material for: Abiotic stress responses in plants: roles of calmodulin-regulated proteins
Source: Front Plant Sci. 2015 Oct 14;6:809. doi: 10.3389/fpls.2015.00809 (PMC4604306; doi:10.3389/fpls.2015.00809)
Supplement: Supplementary file 6 [file Table6.DOC]

| **Table S6:** Homology analysis of mitogen-activated protein kinase phosphatase 1 (MKP1) of different plants. | | | | | | | | | | | | | | | | |
| --- | --- | --- | --- | --- | --- | --- | --- | --- | --- | --- | --- | --- | --- | --- | --- | --- |
|  | **1** | **2** | **3** | **4** | **5** | **6** | **7** | **8** | **9** | **10** | **11** | **12** | **13** | **14** | **15** | **16** |
| **1. TMKP1** | **Similarity**  **Identity** | 80.3 | 49.5 | 43.5 | 49.5 | 86.1 | 78.9 | 78.5 | 68.6 | 47.9 | 47.9 | 80.5 | 49 | 51 | 49.1 | 49.8 |
| **2. OsMKP1** | 87.4 |  | 49 | 42.9 | 49.4 | 82.1 | 82.4 | 80.3 | 70.3 | 47.9 | 47.7 | 84.9 | 49.3 | 51.5 | 49.5 | 49.9 |
| **3. AtMKP1** | 65.1 | 66.6 |  | 40.7 | 58.9 | 49.5 | 50.1 | 49.3 | 46.1 | 53.7 | 52.8 | 49.5 | 57.7 | 59.8 | 57.4 | 57.7 |
| **4. NtMKP1** | 57.5 | 57.1 | 59.2 |  | 42.8 | 42.8 | 43.4 | 43.4 | 39.3 | 40.8 | 40.6 | 44 | 41.7 | 41.5 | 41.8 | 42.1 |
| **5. GrMKP1** | 63.5 | 62.6 | 73.1 | 61.1 |  | 51.2 | 49.8 | 49.7 | 44.9 | 63.5 | 61.9 | 50 | 71.5 | 71.1 | 71.3 | 72.1 |
| **6. BdMKP1** | 92.3 | 88.4 | 64.7 | 55.8 | 63.1 |  | 79.3 | 78.1 | 69 | 48.6 | 48.4 | 81.6 | 48.5 | 51.2 | 48.9 | 49.3 |
| **7. ZmMKP1.0** | 86.7 | 88.6 | 66.7 | 58.2 | 63.3 | 87.5 |  | 88.8 | 75.8 | 50.6 | 50.1 | 89.7 | 49.4 | 51.2 | 49 | 49.4 |
| **8. ZmMKP1.1** | 85.5 | 87.6 | 66.6 | 57.8 | 64 | 86.2 | 92.7 |  | 84.2 | 49.4 | 48.9 | 88 | 49.4 | 50.9 | 49.3 | 48.7 |
| **9. ZmMKP1.2** | 75.1 | 76 | 59.3 | 52.4 | 58.3 | 75.7 | 79.6 | 84.5 |  | 45.7 | 45.2 | 75 | 45.6 | 48.1 | 45.8 | 45.1 |
| **10. CaMKP1.1** | 60.9 | 61.3 | 68.1 | 59.9 | 75.4 | 61.1 | 62.4 | 62.5 | 56.1 |  | 97.9 | 50 | 62.8 | 61.7 | 62.7 | 62.3 |
| **11. CaMKP1.2** | 61.7 | 62 | 68.3 | 59.3 | 74.9 | 61.7 | 63 | 63 | 56.5 | 97.9 |  | 49.6 | 61.6 | 60.4 | 61.4 | 61.1 |
| **12. SiMKP1** | 87.5 | 89.8 | 66.3 | 57.8 | 62.9 | 88.6 | 93.3 | 92 | 79.3 | 62.2 | 63 |  | 50.5 | 52.4 | 50.1 | 50.1 |
| **13. PtMKP1.0** | 62.6 | 63 | 71.2 | 61.5 | 81.9 | 61.1 | 62.6 | 62.5 | 57.1 | 74.7 | 74.5 | 63.2 |  | 84.1 | 98 | 87.2 |
| **14. PtMKP1.1** | 65.5 | 67.4 | 75.4 | 59.5 | 79.6 | 65.1 | 67.3 | 66.4 | 60.6 | 73.2 | 73 | 66.9 | 87.1 |  | 84.1 | 92.2 |
| **15. PeMKP1.0** | 62.6 | 63.3 | 71.3 | 61 | 81.6 | 61.1 | 61.9 | 62.5 | 56.9 | 74.6 | 74.4 | 62.9 | 98.8 | 87.2 |  | 87.1 |
| **16. PeMKP1.1** | 62.6 | 63.7 | 71.6 | 60.8 | 81.9 | 62 | 63.2 | 63 | 56.7 | 75.1 | 75 | 63.7 | 91.6 | 92.7 | 91.6 |  |

*Triticum turgidum* (TMKP1: ACB05479.1); *Oryza sativa* (OsMKP1: BAF46959.1); *Arabidopsis thaliana* (AtMKP1: AEE79361.1); *Nicotiana tabacum* (NtMKP1: BAD00043.1); *Gossypium* *raimondii* (GrMKP1: XP_012459204.1); *Brachypodium distachyon* (BdMKP1: XP_003569010.1); *Zea mays* (ZmMKP1.0: XP_008648619.1, ZmMKP1.1: XP_008656547.1, ZmMKP1.2: XP_008656548.1); *Cicer arietinum* (CaMKP1.1: XP_004492084.1, CaMKP1.2: XP_012568899.1); *Setaria italica* (SiMKP1: XP_004960490.1); *Populus trichocarpa* (PtMKP1.0: XP_002316314.2, PtMKP1.1: XP_002311140.1), *Populus euphratica* (PeMKP1.0: XP_011027115.1, PeMKP1.1: XP_011021895.1).
