# Supplementary material for: Abiotic stress responses in plants: roles of calmodulin-regulated proteins
Source: Front Plant Sci. 2015 Oct 14;6:809. doi: 10.3389/fpls.2015.00809 (PMC4604306; doi:10.3389/fpls.2015.00809)
Supplement: Supplementary file 7 [file Table7.DOC]

| **Table S7:**  Identity and similarity analysis of calmodulin (CaM)-binding domain of mitogen-activated protein kinase phosphatase 1  (MKP1) of different plants. | | | | | | | | | | | | | | | | |
| --- | --- | --- | --- | --- | --- | --- | --- | --- | --- | --- | --- | --- | --- | --- | --- | --- |
| **CaMBD 1** | **1** | **2** | **3** | **4** | **5** | **6** | **7** | **8** | **9** | **10** | **11** | **12** | **13** | **14** | **15** | **16** |
| **1. TMKP1** | **Similarity**  **Identity** | 79.2 | 42.0 | 38.9 | 45.1 | 83.1 | 74.0 | 75.3 | 75.3 | 46.7 | 46.7 | 76.6 | 45.5 | 45.5 | 45.6 | 45.5 |
| **2. OsMKP1** | 87.0 |  | 39.8 | 35.8 | 44.9 | 83.1 | 87.0 | 88.3 | 88.3 | 46.7 | 46.7 | 87.0 | 43.2 | 43.2 | 42.0 | 43.2 |
| **3. AtMKP1** | 60.3 | 59.0 |  | 38.9 | 58.6 | 37.1 | 37.5 | 38.6 | 38.6 | 50.0 | 50.0 | 39.8 | 57.5 | 55.2 | 57.5 | 55.2 |
| **4. NtMKP1** | 49.5 | 46.3 | 49.5 |  | 45.8 | 34.7 | 33.7 | 34.7 | 34.7 | 43.3 | 43.3 | 35.8 | 45.3 | 45.3 | 45.3 | 45.3 |
| **5. GrMKP1** | 63.2 | 56.3 | 69.0 | 64.2 |  | 44.9 | 41.6 | 43.8 | 43.8 | 68.9 | 68.9 | 46.1 | 83.9 | 82.8 | 83.9 | 81.6 |
| **6. BdMKP1** | 89.6 | 94.8 | 57.7 | 47.4 | 58.6 |  | 80.5 | 81.8 | 81.8 | 42.2 | 42.2 | 83.1 | 38.6 | 40.9 | 38.6 | 40.9 |
| **7. ZmMKP1.0** | 84.4 | 94.8 | 55.1 | 43.2 | 55.2 | 94.8 |  | 94.8 | 94.8 | 43.3 | 43.3 | 93.5 | 43.2 | 40.9 | 40.9 | 40.9 |
| **8. ZmMKP1.1** | 84.4 | 94.8 | 55.1 | 44.2 | 55.2 | 94.8 | 97.4 |  | 100.0 | 45.6 | 45.6 | 94.8 | 44.3 | 42.0 | 42.0 | 42.0 |
| **9. ZmMKP1.2** | 84.4 | 94.8 | 55.1 | 44.2 | 55.2 | 94.8 | 97.4 | 100.0 |  | 45.6 | 45.6 | 94.8 | 44.3 | 42.0 | 42.0 | 42.0 |
| **10. CaMKP1.1** | 56.7 | 56.7 | 63.3 | 61.1 | 82.2 | 54.4 | 55.6 | 55.6 | 55.6 |  | 100.0 | 48.9 | 66.7 | 65.6 | 66.7 | 65.6 |
| **11. CaMKP1.2** | 56.7 | 56.7 | 63.3 | 61.1 | 82.2 | 54.4 | 55.6 | 55.6 | 55.6 | 100.0 |  | 48.9 | 66.7 | 65.6 | 66.7 | 65.6 |
| **12. SiMKP1** | 85.7 | 96.1 | 56.4 | 45.3 | 57.5 | 96.1 | 98.7 | 98.7 | 98.7 | 56.7 | 56.7 |  | 45.5 | 43.2 | 43.2 | 43.2 |
| **13. PtMKP1.0** | 60.9 | 57.5 | 69.0 | 61.1 | 88.5 | 54.0 | 56.3 | 56.3 | 56.3 | 78.9 | 78.9 | 58.6 |  | 89.7 | 97.7 | 88.5 |
| **14. PtMKP1.1** | 59.8 | 56.3 | 65.5 | 56.8 | 85.1 | 55.2 | 55.2 | 55.2 | 55.2 | 75.6 | 75.6 | 57.5 | 92.0 |  | 88.5 | 98.9 |
| **15. PeMKP1.0** | 58.6 | 56.3 | 69.0 | 61.1 | 87.4 | 52.9 | 54.0 | 54.0 | 54.0 | 77.8 | 77.8 | 56.3 | 97.7 | 90.8 |  | 87.4 |
| **16. PeMKP1.1** | 59.8 | 56.3 | 65.5 | 56.8 | 85.1 | 55.2 | 55.2 | 55.2 | 55.2 | 75.6 | 75.6 | 57.5 | 92.0 | 100.0 | 90.8 |  |
| **CaMBD 2** |  |  |  |  |  |  |  |  |  |  |  |  |  |  |  |  |
| **1. TMKP1** |  | 72.0 | 44.0 | 34.6 | 56.0 | 84.0 | 80.0 | 59.3 | 12.0 | 33.3 | 33.3 | 63.0 | 42.3 | 33.3 | 42.3 | 33.3 |
| **2. OsMKP1** | 76.0 |  | 44.0 | 34.6 | 64.0 | 72.0 | 80.0 | 63.0 | 8.0 | 37.0 | 37.0 | 66.7 | 38.5 | 33.3 | 38.5 | 33.3 |
| **3. AtMKP1** | 60.0 | 60.0 |  | 26.9 | 48.0 | 44.0 | 48.0 | 37.0 | 4.0 | 37.0 | 37.0 | 40.7 | 50.0 | 44.4 | 50.0 | 44.4 |
| **4. NtMKP1** | 52.0 | 44.0 | 52.0 |  | 23.1 | 34.6 | 42.3 | 42.3 | 8.3 | 26.9 | 26.9 | 42.3 | 24.0 | 23.1 | 20.0 | 23.1 |
| **5. GrMKP1** | 64.0 | 72.0 | 68.0 | 44.0 |  | 52.0 | 60.0 | 40.7 | 4.0 | 37.0 | 37.0 | 44.4 | 42.3 | 44.4 | 46.2 | 44.4 |
| **6. BdMKP1** | 88.0 | 76.0 | 56.0 | 40.0 | 60.0 |  | 84.0 | 63.0 | 12.0 | 37.0 | 37.0 | 66.7 | 30.8 | 25.9 | 30.8 | 25.9 |
| **7. ZmMKP1.0** | 88.0 | 88.0 | 68.0 | 52.0 | 72.0 | 88.0 |  | 77.8 | 8.0 | 40.7 | 40.7 | 81.5 | 34.6 | 29.6 | 34.6 | 29.6 |
| **8. ZmMKP1.1** | 72.0 | 72.0 | 60.0 | 56.0 | 60.0 | 72.0 | 84.0 |  | 8.0 | 48.0 | 48.0 | 96.0 | 34.6 | 32.0 | 34.6 | 32.0 |
| **9. ZmMKP1.2** | 16.0 | 16.0 | 8.0 | 16.7 | 4.0 | 16.0 | 12.0 | 12.0 |  | 12.0 | 12.0 | 4.0 | 8.0 | 4.0 | 8.0 | 4.0 |
| **10. CaMKP1.1** | 48.0 | 44.0 | 52.0 | 48.0 | 52.0 | 48.0 | 52.0 | 60.0 | 24.0 |  | 100.0 | 52.0 | 46.2 | 48.0 | 46.2 | 48.0 |
| **11. CaMKP1.2** | 48.0 | 44.0 | 52.0 | 48.0 | 52.0 | 48.0 | 52.0 | 60.0 | 24.0 | 100.0 |  | 52.0 | 46.2 | 48.0 | 46.2 | 48.0 |
| **12. SiMKP1** | 76.0 | 76.0 | 64.0 | 56.0 | 64.0 | 76.0 | 88.0 | 96.0 | 8.0 | 64.0 | 64.0 |  | 38.5 | 36.0 | 38.5 | 36.0 |
| **13. PtMKP1.0** | 64.0 | 56.0 | 60.0 | 56.0 | 68.0 | 56.0 | 64.0 | 60.0 | 12.0 | 60.0 | 60.0 | 64.0 |  | 80.8 | 96.0 | 80.8 |
| **14. PtMKP1.1** | 56.0 | 52.0 | 56.0 | 48.0 | 68.0 | 48.0 | 56.0 | 56.0 | 16.0 | 60.0 | 60.0 | 60.0 | 88.0 |  | 84.6 | 100.0 |
| **15. PeMKP1.0** | 64.0 | 56.0 | 64.0 | 52.0 | 72.0 | 56.0 | 64.0 | 60.0 | 12.0 | 64.0 | 64.0 | 64.0 | 96.0 | 92.0 |  | 84.6 |
| **16. PeMKP1.1** | 56.0 | 52.0 | 56.0 | 48.0 | 68.0 | 48.0 | 56.0 | 56.0 | 16.0 | 60.0 | 60.0 | 60.0 | 88.0 | 100.0 | 92.0 |  |

**Similarity**

**Identity**

*Triticum turgidum* (TMKP1: ACB05479.1); *Oryza sativa* (OsMKP1: BAF46959.1); *Arabidopsis thaliana* (AtMKP1: AEE79361.1); *Nicotiana tabacum* (NtMKP1: BAD00043.1); *Gossypium* *raimondii* (GrMKP1: XP_012459204.1); *Brachypodium distachyon* (BdMKP1: XP_003569010.1); *Zea mays* (ZmMKP1.0: XP_008648619.1, ZmMKP1.1: XP_008656547.1, ZmMKP1.2: XP_008656548.1); *Cicer arietinum* (CaMKP1.1: XP_004492084.1, CaMKP1.2: XP_012568899.1); *Setaria italica* (SiMKP1: XP_004960490.1); *Populus trichocarpa* (PtMKP1.0: XP_002316314.2, PtMKP1.1: XP_002311140.1), and *Populus euphratica* (PeMKP1.0: XP_011027115.1, PeMKP1.1: XP_011021895.1).
