# Supplementary figures and images for: Abiotic stress responses in plants: roles of calmodulin-regulated proteins
Source: Front Plant Sci. 2015 Oct 14;6:809. doi: 10.3389/fpls.2015.00809 (PMC4604306; doi:10.3389/fpls.2015.00809)

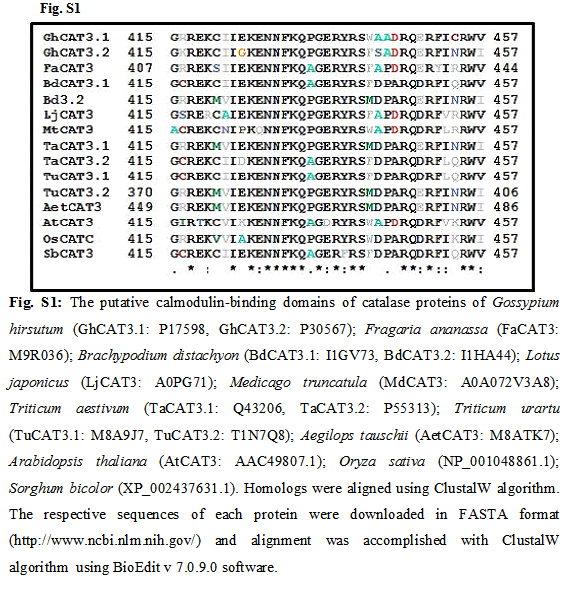

Supplement: Supplementary file 8 [file Image1.JPEG]

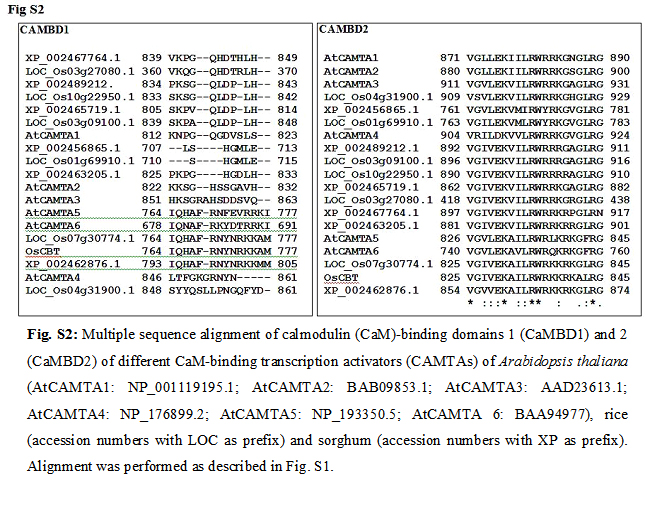

Supplement: Supplementary file 9 [file Image2.JPEG]
