# Supplementary material for: Abiotic stress responses in plants: roles of calmodulin-regulated proteins
Source: Front Plant Sci. 2015 Oct 14;6:809. doi: 10.3389/fpls.2015.00809 (PMC4604306; doi:10.3389/fpls.2015.00809)
Supplement: Supplementary file 10 [file Image3.PDF]

```
TMKP1     1   MANPDDGG---------------GRKFWRSASWSAPR------AAEAGPEAAP----------LPPRM 37
OsMKP1    1   MATPDDGGGGVGGG---------AGKKFWRSASWSASR---DTPPDAATPAGAGGGGAGA----GQARR 54
AtMKP1    1   MVGREDAMGNDEAP----PGS---KKMFWRSASWSASRTASQVPEGDEQSLNIPCAISS-------GPSR 56
NtMKP1    1   MLGVDEKDRVPGGN----------RKTYARSVSWSDRS-------------------------------- 28
GrMKP1    1   MVGKEDPPASPRAPSCQLSSS---RKMFWRSASWSSSRTSGQIPLTEDKDLGAGSNGNDGINN---GQTR 64
BdMKP1    1   MANPDDGGA----A---------AGKKFWRSASWSASR------VGEAAKEGAVGGG--------GPARL 43
ZmMKP1.0  1   MATPDDGPAPAGPG---------V-RKFWRSASWSEPR---EPLPDAAAGPGAAGG----------RSRR 47
ZmMKP1.1  1   MATPDDGPAPAGG-----------RKFWRSVSWSEAR---EPPP--PAPPDVAGG----------QSRR 43
ZmMKP1.2  1   MATPDDGPAPAGG-----------RKFWRSVSWSEAR---EPPP--PAPPDVAGG----------QSRR 43
CaMKP1.1  1   MVGNDDAR----AP-CHLSSS---RKMFWRSASWSSARSSAANPSGEPESNFADPNSGPENSN------R 56
CaMKP1.2  1   -------------------------MFWRSASWSSARSSAANPSGEPESNFADPNSGPENSN------R 38
SiMKP1    1   MATPDDGPAAAGGG----------RKFWRSASWSASRAAAEPPQDGAAPG--AGG----------QARR 47
PtMKP1.0  1   MVDKEDASNNPLAP-CQLPSAGGSRKVFWRSVSWSSSRTASQNPGTEEQDRVIDPNGNPAGNNSGSGQNR 69
PtMKP1.1  1   MVGKEDASNNPLAP-CQLPSAGGSRKVFWRSASWSSSRTALQHPGTEEKDCVIDPNGNPAGNNSGNGQNR 69
PeMKP1.0  1   MVDKEDASNNPLAP-CQLPSAGGSRKVFWRSVSWSSSRTASENPGTEEKDRVIDPNGNPAGNNSGNGQNR 69
PeMKP1.1  1   MVGKEDASNNPLAP-SQLPSAGGSRKVFWRSASWSSSRTALQHPGTEEKDCVIDPNGNPAGNNSGNGQNR 69

TMKP1     38  GPPP--LTPRS----KGR-ACLPPLQPLAITRRSLDEWPKAGSDDVGEWPNPT-TPGASKAGGGGGGPGS 99
OsMKP1    55  IPPPPPLTPR---GGKGR-SCLPPLQPLNITRRSLDEWPRAGSDDVGEWPNPT-TPGASKAEGAG----S 115
AtMKP1    57  RCPAAPLTPRSHHNSKAR-ACLPPLQPLAISRRSLDEWPKAGSDDVGEWPHPP-TPSG------------ 112
NtMKP1    28  ---PTKSSAKPQWNSKAR-ACLPPLQPLSITRPTAEEWPCAGSDDLGVWPNNPPTPGVRLGPVS------ 88
GrMKP1    65  RFPPPPLTPRSQQNCKAR-SCLPPLQPLSIARRSLDEWPKAGSDDLGEWPQPP-TPSG------------ 120
BdMKP1    44  VPPPPPLTPRS----KGR-SCLPPLQPLAITRRSLDEWPKAGSDDVGEWPNPT-TPGASKAGCGG--PAS 105
ZmMKP1.0  48  VPPPPPLTPRSM-SSKAR-SCLPPLQPLAITRRSLDEWPRAGSDDVGEWPNPT-TPGASKAGGG---PSS 111
ZmMKP1.1  44  GLPPPPLTPRSM-SSKAR-SCLPPLQPLAITRRSLDEWPKAGSDDVGEWPNPT-TPGASRAGGGGGGSSP 110
ZmMKP1.2  44  GLPPPPLTPRSM-SSKAR-SCLPPLQPLAITRRSLDEWPKAGSDDVGEWPNPT-TPGASRAGGGGGGSSP 110
CaMKP1.1  57  RFPPPPLTPRSQQNCKARSSCLPPLQPLSIARRSLDEWPKASSDDIGEWPQPPTTPSGRGNNSNC----- 121
CaMKP1.2  39  RFPPPPLTPRSQQNCKARSSCLPPLQPLSIARRSLDEWPKASSDDIGEWPQPPTTPSGRGNNSNC----- 103
SiMKP1    48  VPPPPPLTPRSM-SAKAR-SCLPPLQPLAITRRSLDEWPKAGSDDVGEWPNPT-TPGASKVDGG---PSS 111
PtMKP1.0  70  RYPAP-LTPRSQQNSKAR-SCLPPLQPLSIARRSVDEWPKASSDDLGEWPQPP-TPSG------------ 124
PtMKP1.1  70  RYPAP-LTPRSQQNCKAR-SCLP---PLSIARRSLDEWPKAGSDDSGEWPQPP-TPSG------------ 121
PeMKP1.0  70  RYPAP-LTPRSQQNSKAR-SCLPPLQPLSIARRSVDEWPKASSDDLGEWPQPP-TPSG------------ 124
PeMKP1.1  70  RYPAP-LTPRSQQNCKAR-SCLP---PLSIARKSLDEWPKAGSDDLGEWPQPP-TPSG------------ 121

TMKP1     100 AKPGEGLRLDLSTLRSQ-------------GRKDQIAFFDKECSKVAEHVYLGGDAVAKNRDILRKNGI 155
OsMKP1    116 AKPGEGLRLDLSSLRSQ-------------GRKDQIAFFDKECSKVADHVYLGGDAVAKNRDILRKNGI 171
AtMKP1    113 NKTGERLKLDLSSTQQR------VTDKSSGLAKREKIAFFDKECSKVADHIYVGGDAVAKDKSILKNNGI 176
NtMKP1    89  THVGSATKRVPREFEFK---------------KDKLAFFNKECSKIVDHIYLGSDTVAKNRDILHENGI 142
GrMKP1    121 NKSGERLKLDLSSIQRN-------NDKNGGLVKRDKIAFFDKECSKVAEHIYLGGDAVAKDREILKKNGI 183
BdMKP1    106 AKPGEGLRLDLSTLRSQ-------------GRKDQIAFFDKECSKVAEHVYLGGDAVAKNREILRKNGI 161
ZmMKP1.0  112 ARPGEGLRLDLSSLRSQ-------------GRKDQIAFFDKECSKVADHVYLGGDAVAKNRDILRKNGI 167
ZmMKP1.1  111 AKPGEGLRLDLSSLRSQ-------------GRKDQIAFFDKECSKVADHVYLGGDAVAKNRDILRKNGI 166
ZmMKP1.2  111 AKPGEGLRLDLSSLRSQ-------------GRKDQIAFFDKECSKVADHVYLGGDAVAKNRDILRKNGI 166
CaMKP1.1  122 NSNGERLKLDLSQIQQNNINNNHDSRNNGGLVKRDKIAFFDKECSKVAEHVYLGGDAVAKDREILKRNGI 191
CaMKP1.2  104 NSNGERLKLDLSQIQQNNINNNHDSRNNGGLVKRDKIAFFDKECSKVAEHVYLGGDAVAKDREILKRNGI 173
SiMKP1    112 AKPGEGLRLDLSSLRMQ-------------GRKDQIAFFDKECSKVAEHVYLGGDAVAKNRDILRKNGI 167
PtMKP1.0  125 NKSGERLKLDLSSIQRN-------PDRNVGLVKKDRIAFFGKECSKVAEHVYLGGDAVAREREILKQNGI 187
PtMKP1.1  122 NKSGERLKLDLSSIQRT-------PDRNGGLVKKDRIAFFDKECSKVAEHVYLGGDAVARDREILKQNGI 184
PeMKP1.0  125 NKSGERLKLDLSSIQRN-------PDRNVGLVKKDRIAFFGKECSKVAEHVYLGGDAVAREREILKQNGI 187
PeMKP1.1  122 NKSGERLKLDLSSIQRT-------PDRNGGLVKKDRIAFFDKECSKVAEHVYLGGDAVAKDREILKQNGI 184
```

Dual specificity phosphatases (DSP)

```
TMKP1     156 THVLNCVGFVCPEYFKSDLVYRTLWLQDSPTEDITSILYDVFDYFEDVREQAGRVFVHCCQGVSRSTSLV 225
OsMKP1    172 THVLNCVGFVCPEYFKSDLVYRTLWLQDSPTEDITSILYDVFDYFEDVREQGGRVFVHCCQGVSRSTSLV 241
AtMKP1    177 THILNCVGFICPEYFKSDFCYRSLWLQDSPSEDITSILYDVFDYFEDVREQSGRIFVHCCQGVSRSTSLV 246
NtMKP1    143 THVLNCVGFSCPEYFKDDLVYKTLWLQDSPTEDITSILYDVFDYFEDVREQGGRVFVHCYQGVSRSTSLV 212
GrMKP1    184 THVLNCVGFVCPEYFKADFVYRTLWLQDSPSEDITSILYDVFDYFEDVRELGGRVFVHCCQGVSRSTSLV 253
BdMKP1    162 THVLNCVGFVCPEYFKSDLVYRTLWLQDSPTEDITSILYDVFDYFEDVREQSGRVFVHCCQGVSRSTSLV 231
ZmMKP1.0  168 THVLNCVGFVCPEYFKSDLVYRTLWLQDSPTEDITSILYDVFDYFEDVREQGGRVFVHCCQGVSRSTSLV 237
ZmMKP1.1  167 THVLNCVGFVCPEYFKSDLVYRTLWLQDSPTEDITSILYDVFDYFEDVREQGGRVLVHCCQGVSRSTSLV 236
ZmMKP1.2  167 THVLNCVGFVCPEYFKSDLVYRTLWLQDSPTEDITSILYDVFDYFEDVREQGGRVLVHCCQGVSRSTSLV 236
CaMKP1.1  192 THVLNCVGFVCPEYFKADFVYRTLWLQDSPSEDITSILYDVFDYFEDVREKNGRVFVHCCQGVSRSTSLV 261
CaMKP1.2  174 THVLNCVGFVCPEYFKADFVYRTLWLQDSPSEDITSILYDVFDYFEDVREKNGRVFVHCCQGVSRSTSLV 243
SiMKP1    168 THVLNCVGFVCPEYFKSDLVYRTLWLQDSPTEDITSILYDVFDYFEDVREQGGRVFVHCCQGVSRSTSLV 237
PtMKP1.0  188 THVLNCVGFVCPEYFKADFVYRTLWLQDSPSEDITSILYDVFDYFEDVREQGGRVFVHCCQGVSRSTSLV 257
PtMKP1.1  185 THVLNCVGFVCPEYFKADFVYRTLWLQDSPSEDITSILYDVFDYFEDVREQGGRVFVHCCQGVSRSTSLV 254
PeMKP1.0  188 THVLNCVGFVCPEYFKADFVYRTLWLQDSPSEDITSILYDVFDYFEDVREQGGRVFVHCCQGVSRSTSLV 257
PeMKP1.1  185 THVLNCVGFVCPEYFKADFVYRTLWLQDSPSEDITSILYDVFDYFEDVREQGGRVFVHCCQGVSRSTSLV 254
```

DSP   Protein tyrosine phosphatase 184-248

```
TMKP1     226 IAYLMWREGQSFDDAFQFVKAARGIANPNMGFACQLLQCQKRVHAIPLSPNSVLRMYRLAPHSPYAPLHL 295
OsMKP1    242 IAYLMWREGQSFDDAFQLVKAARGIANPNMGFACQLLQCQKRVHAIPLSPNSVLRMYRMAPHSPYAPLHL 311
AtMKP1    247 IAYLMWREGQSFDDAFQYVKSARGIADPNMGFACQLLQCQKRVHAFPLSPTSLLRMYKMSPHSPYDPLHL 316
NtMKP1    213 IAYLMWKEGLSFEDAFQHVKAARGVTNPNMGFACQLLQCQKRVHALPVSPSSVLRMYRMAPHSPYDPLHL 282
GrMKP1    254 IAYLMWREGQSFDDAFQYVKAARGIADPNMGFACQLLQCQKRVHAFPLSPSSLLRMYRIAPHSPYDPLHL 323
BdMKP1    232 IAYLMWREGQSFDDAFQFVKAARGIANPNMGFACQLLQCQKRVHAIPLSPNSVLRMYRMAPHSPYAPLHL 301
ZmMKP1.0  238 IAYLMWREGQSFDDAFQFVKAARGIANPNMGFACQLLQCQKRVHAIPLSPNSVLRMYRMAPHSQYAPLHL 307
ZmMKP1.1  237 IAYLMWREGQSFDDAFQFVKAARGIANPNMGFACQLLQCQKRVHAIPLSPNSVLRMYRMAPHSQYAPLHL 306
ZmMKP1.2  237 IAYLMWREGQSFDDAFQFVKAARGIANPNMGFACQLLQCQKRVHAIPLSPNSVLRMYRMAPHSQYAPLHL 306
CaMKP1.1  262 IAYLMWREGQSFDDAFQFVKAARGIADPNMGFACQLLQCQKRVHAFPLSPSSLLRMYRIAPHSPYDPLHL 331
CaMKP1.2  244 IAYLMWREGQSFDDAFQFVKAARGIADPNMGFACQLLQCQKRVHAFPLSPSSLLRMYRIAPHSPYDPLHL 313
SiMKP1    238 IAYLMWREGQSFDDAFQFVKAARGIANPNMGFACQLLQCQKRVHAIPLSPNSVLRMYRMAPHSQYAPLHL 307
PtMKP1.0  258 IAYLMWREGQSFDDAFQYVKAARGIADPNMGFACQLLQCQKRVHAFPLSPSSLLRMYRIAPHSPYDPLHL 327
PtMKP1.1  255 IAYLMWREGQSFDDAFQYVKAARGIADPNMGFACQLLQCQKRVHAFPLSPSSLLRMYRIAPHSPYDPLHL 324
PeMKP1.0  258 IAYLMWREGQSFDDAFQYVKAARGIADPNMGFACQLLQCQKRVHAFPLSPSSLLRMYRIAPHSPYDPLHL 327
PeMKP1.1  255 IAYLMWREGQSFDDAFQYVKAARGIADPNMGFACQLLQCQKRVHAFPLSPSSLLRMYRIAPHSSYDPLHL 324
```

Protein tyrosine phosphatase 184-248   DSP

```
TMKP1     296 VPKMLNEPSPAALDSRGAFIVHVLSSIYVWVGIKCDTVMEKDARAAAFQVVRYEKVQGQIKVVREGLEQP 365
OsMKP1    312 VPKMLNEPSPAALDSRGAFIVHVLSSIYVWVGMKCDQVMEKDARAAAFQVVRYEKVQGHIKVVREGSEVP 381
AtMKP1    317 VPKLLNDPCPGSLDSRGAFIIQLPSAIYIWVGRQCETIMEKDAKAAVCQIARYEKVEAPIMVVREGDEPV 386
NtMKP1    283 VPKMLNEAGAEGFDSRGAFLVLIPSAIYVWIGKHCDSVMSDNARAAAFQVIRYEKAQGPVIIINEGKEPS 352
GrMKP1    324 VPKMLNDPSPSGLDSRGAFIVHIPSAIYIWIGKNCESIMERDVRGAVCQIVRYERVQGPVIVIKEGEEPA 393
BdMKP1    302 VPKMLNEPSPAALDSRGAFIVHIASLIYVWVGLRCDQVMEKDARAAAFQVVRYEKVQGHIKVVREGLEQP 371
ZmMKP1.0  308 VPKMLNDPSPAALDSRGAFIVHVLLSIYVWVGMKCDPVMEKDAKAAAFQVVRYEKVQGHIKVVREGLEPQ 377
ZmMKP1.1  307 VPKMLNDPSPATLDSRGAFIVHVLSSLYVWVGMKCDPVMEKDAKAAAFQVVRYEKVQGHIKVVREGLEPQ 376
ZmMKP1.2  307 VPKMLNDPSPATLDSRGAFIVHVLSSLYVWVGMKCDPVMEKDAKAAAFQVVRYEKVQGHIKVVREGLEPQ 376
CaMKP1.1  332 VPKMLTDPSSNALDSRGAFIVHIPSAIYVWIGKNCEAIMERDARGAAGQIVRYERVQGSIIMIKEGEEPL 401
CaMKP1.2  314 VPKMLTDPSSNALDSRGAFIVHIPSAIYVWIGKNCEAIMERDARGAAGQIVRYERVQGSIIMIKEGEEPL 383
SiMKP1    308 VPKMLNEPSPAALDSRGAFIVHVLSSIYVWVGMKCDPVMEKDARAAAFQVVRYEKVQGHIKVVREGLEQQ 377
PtMKP1.0  328 VPRMLNDPSLSALDSRGAFIVHIPSSIYVWIGKNCEAIMERDARGAVCQIVRYERALGPIIVVKEGEEPA 397
PtMKP1.1  325 VPRMLNDPSPSALDSRGAFIVHIPSSIYVWIGKNCEAIMERDARGAVCQIVRYERAQGPIIVVKEGEEPA 394
PeMKP1.0  328 VPRMLNDPSLSALDSRGAFIVHIPSSIYVWIGKNCEAIMERDARGAVCQIVRYERALGPIIVVKEGEEPA 397
PeMKP1.1  325 VPRMLNDPSPSALDSRGAFIVHIPSSIYVWIGKNCEAIMERDARGAACQIVRYERAQGSIIVVKEGEEPA 394
```

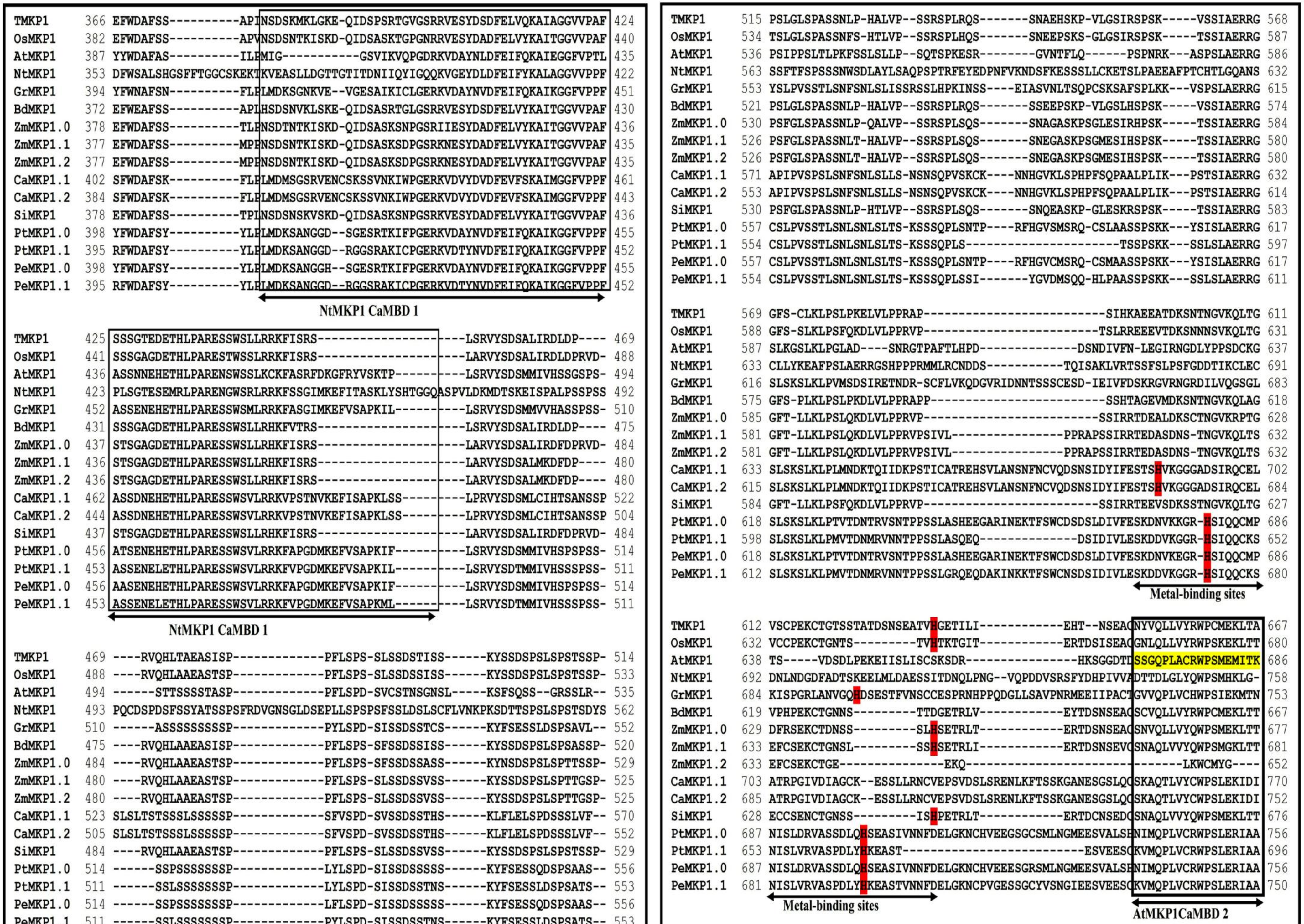

TMKP1 366 EFWDAFSS----------APINSDSKMKLGKE-QIDSPSRTGVGSRRVESYDSDFELVQKAIAGGVVPAF 424
OsMKP1 382 EFWDAFSS----------APVNSDSNTKISKD-QIDSASKTGPGNRRVESYDADFELVYKAITGGVVPAF 440
AtMKP1 387 YYWDAFAS----------ILEMIG-----------GSVIKVQPGDRKVDAYNLDFEIFQKAIEGGFVPTL 435
NtMKP1 353 DFWSALSHGSFFTGGCSKEKTKVEASLLDGTTGTITDNIIQYIGQQKVGEYDLDFEIFYKALAGGVVPPF 422
GrMKP1 394 YFWNAFSN----------FLPLMDKSGNKVE--VGESAIKICLGERKVDAYNVDFEIFQKAIKGGFVPPF 451
BdMKP1 372 EFWEAFSS----------APIHSDSNVKLSKE-QIDSASRTGLGSRRVESYDSDFELVYKAITGGVVPAF 430
ZmMKP1.0 378 EFWDAFSS----------TLPNSDTNTKISKD-QIDSASKSNPGSRIIESYDADFELVYKAITGGVVPAF 436
ZmMKP1.1 377 EFWDAFSS----------MPPNSDSNTKISKD-QIDSASKSDPGSRKNESYDADFELVYKAITGGVVPAF 435
ZmMKP1.2 377 EFWDAFSS----------MPPNSDSNTKISKD-QIDSASKSDPGSRKNESYDADFELVYKAITGGVVPAF 435
CaMKP1.1 402 SFWDAFSK----------FLPLMDMSGSRVENCSKSSVNKIWPGERKVDVYDVDFEVFSKAIMGGFVPPF 461
CaMKP1.2 384 SFWDAFSK----------FLPLMDMSGSRVENCSKSSVNKIWPGERKVDVYDVDFEVFSKAIMGGFVPPF 443
SiMKP1 378 EFWDAFSS----------TPINSDSNSKVSKD-QIDSASKSNPGSRKVESYDADFELVYKAITGGVVPAF 436
PtMKP1.0 398 YFWDAFSY----------YLPLMDKSANGGD--SGESRTKIFPGERKVDAYNVDFEIFQKAIKGGFVPPF 455
PtMKP1.1 395 RFWDAFSY----------YLPLMDKSANGGD--RGGSRAKICPGERKVDTYNVDFEIFQKAIKGGFVPPF 452
PeMKP1.0 398 YFWDAFSY----------YLPLMDKSANGGH--SGESRTKIFPGERKVDAYNVDFEIFQKAIKGGFVPPF 455
PeMKP1.1 395 RFWDAFSY----------YLPLMDKSANGGD--RGGSRAKICPGERKVDTYNVDFEIFQKAIKGGFVPPF 452
NtMKP1 CaMBD 1
TMKP1 425 SSSGTEDETHLPARESSWSLLRRKFISRS---------------------LSRVYSDSALIRDLDP---- 469
OsMKP1 441 SSSGAGDETHLPARESTWSSLRRKFISRS---------------------LARVYSDSALIRDLDPRVD- 488
AtMKP1 436 ASSNNEHETHLPARENSWSSLKCKFASRFDKGFRYVSKTP-----------LSRVYSDSMMIVHSSGGSPS- 494
NtMKP1 423 PLSGTESEMRLPARENGWSRLRRKFSSGIMKEFITASKLYSHTGGQASPVLDKMDTSKEISPALPSSPSS 492
GrMKP1 452 ASSENEHETHLPARESSWSMLRRKFASGIMKEFVSAPKIL--------LSRVYSDSMMVVHASSPSS- 510
BdMKP1 431 SSSGAGDETHLPARESSWSLLRHKFVTRS---------------------LSRVYSDSALIRDLDP---- 475
ZmMKP1.0 437 STSGAGDETHLPARESSWSLLRHKFISRS---------------------LARVYSDSALIRDFDPRVD- 484
ZmMKP1.1 436 STSGAGDETHLPARESSWSLLRHKFISRS---------------------LARVYSDSALMKDFDP---- 480
ZmMKP1.2 436 STSGAGDETHLPARESSWSLLRHKFISRS---------------------LARVYSDSALMKDFDP---- 480
CaMKP1.1 462 ASSDNEHETHLPARESSWSVLRRKVPSTNVKEFISAPKLSS--------LPRVYSDSMLCIHTSANSSP 522
CaMKP1.2 444 ASSDNEHETHLPARESSWSVLRRKVPSTNVKEFISAPKLSS--------LPRVYSDSMLCIHTSANSSP 504
SiMKP1 437 STSGAGDETHLPARESSWSLLRHKFISRS---------------------LARVYSDSALIRDFDPRVD- 484
PtMKP1.0 456 ATSENEHETHLPARESSWSVLRRKFAPGDMKEFVSAPKIF--------LSRVYSDSMMIVHSPSPSS- 514
PtMKP1.1 453 ASSENELETHLPARESSWSVLRRKFVPGDMKEFVSAPKIL--------LSRVYSDTMMIVHSSSPSS- 511
PeMKP1.0 456 AASENEHETHLPARESSWSVLRRKFAPGDMKEFVSAPKIF--------LSRVYSDSMMIVHSPSPSS- 514
PeMKP1.1 453 ASSENELETHLPARESSWSVLRRKFVPGDMKEFVSAPKML--------LSRVYSDTMMIVHSSSPSS- 511
NtMKP1 CaMBD 1
TMKP1 469 ----RVQHLTAEASISP-------------PFLSPS-SLSSDSTISS------KYSSDSPSLSPSTSSP- 514
OsMKP1 488 ----RVQHLAAEASTSP-------------PFLSPS-SLSSDSSISS------KYSSDSPSLSPSTSSP- 533
AtMKP1 494 ------STTSSSSTASP-------------PFLSPD-SVCSTNSGNSL-----KSFSQSS--GRSSLR-- 535
NtMKP1 493 PQCDSPDSFSSYATSSPSFRDVGNSGLDSEPLLSPSPSFSSLDSLSCFLVNKPKSDTTSPSLSPSTSDYS 562
GrMKP1 510 ------ASSSSSSSSP-------------PYLSPD-SISSDSSTCS------KYFSESSLDSPSAVL-- 552
BdMKP1 475 ----RVQHLAAEASISP-------------PFLSPS-SFSSDSSISS------KYSSDSPSLSPSASSP- 520
ZmMKP1.0 484 ----RVQHLAAEASTSP-------------PFLSPS-SFSSDSSASS------KYNSDSPSLSPTTSSP- 529
ZmMKP1.1 480 ----RVQHLAAEASTSP-------------PFLSPS-SLSSDSSVSS------KYSSDSPSLSPTTGSP- 525
ZmMKP1.2 480 ----RVQHLAAEASTSP-------------PFLSPS-SLSSDSSVSS------KYSSDSPSLSPTTGSP- 525
CaMKP1.1 523 SLSLTSTSSSLSSSSSP-------------SFVSPD-SVSSDSSTHS------KLFLELSPDSSSLVF-- 570
CaMKP1.2 505 SLSLTSTSSSLSSSSSP-------------SFVSPD-SVSSDSSTHS------KLFLELSPDSSSLVF-- 552
SiMKP1 484 ----RVQHLAAEASTSP-------------PFLSPS-SLSSDSSVSS------KYSSDSPSLSPSTSSP- 529
PtMKP1.0 514 ------SSPSSSSSSSP-------------LYLSPD-SISSDSSSSS------KYFSESSQDSPSAAS-- 556
PtMKP1.1 511 ------SSLSSSSSSSP-------------LYLSPD-SISSDSSTNS------KYFSESSLDSPSATS-- 553
PeMKP1.0 514 ------SSPSSSSSSSP-------------LFLSPD-SISSDSSSSS------KYFSESSQDSPSAAS-- 556
PeMKP1.1 511 ------SSLSSSSSSSP-------------PYLSPD-SISSDSSTNS------KYFSESSLDSPSATS-- 553
TMKP1 515 PSLGLSPASSNLP-HALVP--SSRSPLRQS-------SNAEHSKP-VLGSIRSPSK-----VSSIAERRG 568
OsMKP1 534 TSLGLSPASSNFS-HTLVP--SSRSPLHQS-------SNEEPSKS-GLGSIRSPSK-----TSSIAERRG 587
AtMKP1 536 PSIPPSLTLPKFSSLSLLP--SQTSPKESR-------GVNTFLQ------PSPNRK---ASPSLAERRG 586
NtMKP1 563 SSFTFSPSSSNWSDLAYLSAQPSPTRFEYEDPNFVKNDSFKESSSLLCKETSLPAEEAFPTCHTLGQANS 632
GrMKP1 553 YSLPVSSTLSNFSNLSLISSRSSLHPKINSS----EIASVNLTSQPCSKSAFSPLKK---VSPSLAERRG 615
BdMKP1 521 PSLGLSPASSNLP-HALVP--SSRSPLRQS-------SSEEPSKP-VLGSLHSPSK-----VSSIAERRG 574
ZmMKP1.0 530 PSFGLSPASSNLP-QALVP--SSRSPLSQS-------SNAGASKPSGLESIRHPSK-----TSSIAERRG 584
ZmMKP1.1 526 PSFGLSPASSNLT-HALVP--SSRSPLSQS-------SNEGASKPSGMESIHSPSK-----TSSIAERRG 580
ZmMKP1.2 526 PSFGLSPASSNLT-HALVP--SSRSPLSQS-------SNEGASKPSGMESIHSPSK-----TSSIAERRG 580
CaMKP1.1 571 APIPVSPSLSNFSNLSLLS-NSNSQPVSKCK----NNHGVKLSPHPFSQPAALPLIK---PSTSIAERRG 632
CaMKP1.2 553 APIPVSPSLSNFSNLSLLS-NSNSQPVSKCK----NNHGVKLSPHPFSQPAALPLIK---PSTSIAERRG 614
SiMKP1 530 PSFGLSPASSNLP-HTLVP--SSRSPLSQS-------SNQEASKP-GLESKRSPSK-----TSSIAERRG 583
PtMKP1.0 557 CSLPVSSTLSNLSNLSLTS-KSSSQPLSNTP----RFHGVSMSRQ-CSLAASSPSKK---YSISLAERRG 617
PtMKP1.1 554 CSLPVSSTLSNLSNLSLTS-KSSSQPLS---------------------TSSPSKK---SSLSLAERRG 597
PeMKP1.0 557 CSLPVSSTLSNLSNLSLTS-KSSSQPLSNTP----RFHGVCMSRQ-CSMAASSPSKK---YSISLAERRG 617
PeMKP1.1 554 CSLPVSSTLSNLSNLSLTS-KSSSQPLSSI-------YGVDMSQQ-HLPAASSPSKK---SSLSLAERRG 611
TMKP1 569 GFS-CLKLPSLPKELVLPPRAP------------------------SIHKAEEATDKSNTNGVKQLTG 611
OsMKP1 588 GFS-SLKLPSFQKDLVLPPRVP------------------------TSLRREEEVTDKSNNNSVKQLTG 631
AtMKP1 587 SLKGSLKLPGLAD----SNRGTPAFTLHPD--------------DSNDIVFN-LEGIRNGDLYPPSDCKG 637
NtMKP1 633 CLLYKEAFPSLAERRGSHPPPRMMLRCNDDS----------TQISAKLVRTSSFSLPSFGDDTIKCLEC 691
GrMKP1 616 SLSKSLKLPVMSDSIRETNDR-SCFLVKQDGVRIDNNTSSSCESD-IEIVFDSKRGVRNGRDILVQGSGL 683
BdMKP1 575 GFS-PLKLPSLPKDLVLPPRAPP-----------------------SSHTAGEVMDKSNTNGVKQLAG 618
ZmMKP1.0 585 GFT-LLKLPSLQKDLVLPPRVP------------------------SSIRRTDEALDKSCTNGVKRPTG 628
ZmMKP1.1 581 GFT-LLKLPSLQKDLVLPPRVPSIVL---------------PPRAPSSIRRTEDASDNS-TNGVKQLTS 632
ZmMKP1.2 581 GFT-LLKLPSLQKDLVLPPRVPSIVL---------------PPRAPSSIRRTEDASDNS-TNGVKQLTS 632
CaMKP1.1 633 SLSKSLKLPLMNDKTQIIDKPSTICATREHSVLANSNFNCVQDSNSIDYIFESTSHVKGGGADSIRQCEL 702
CaMKP1.2 615 SLSKSLKLPLMNDKTQIIDKPSTICATREHSVLANSNFNCVQDSNSIDYIFESTSHVKGGGADSIRQCEL 684
SiMKP1 584 GFT-LLKLPSFQKDLVLPPRVP------------------------SSIRRTEEVSDKSSTNGVKQLTG 627
PtMKP1.0 618 SLSKSLKLPTVTDNTRVSNTPPSSLASHEEGARINEKTFSWCDSDSLDIVFESKDNVKKGR-HSIQQCMP 686
PtMKP1.1 598 SLSKSLKLPMVTDNMRVNNTPPSSLASQEQ--------------DSIDIVLESKDDVKGGR-HSIQQCKS 652
PeMKP1.0 618 SLSKSLKLPTVTDNTRVSNTPPSSLASHEEGARINEKTFSWCDSDSLDIVFESKDNVKEGR-HSIQQCMP 686
PeMKP1.1 612 SLSKSLKLPMVTDNMRVNNTPPSSLGRQEQDAKINKKTFSWCNSDSIDIVLESKDDVKGGR-HSIQQCKS 680
Metal-binding sites
TMKP1 612 VSCPEKCTGTSSTATDSNSEATVHGETILI-----------EHT--NSEACNYVQLLVYRWPCMEKLTA 667
OsMKP1 632 VCCPEKCTGNTS---------TVHTKTGIT-----------ERTDSISEACGNLQLLVYRWPSKEKLTT 680
AtMKP1 638 TS-----VDSDLPEKEIISLISCSKSDR---------------HKSGGDTLSSGQPLACRWPSMEMITK 686
NtMKP1 692 DNLNDGDFADTSKEELMLDAESSITDNQLPNG--VQPDDVSRSFYDHPIVVADTTDLGLYQWPSMHKLG- 758
GrMKP1 684 KISPGRLANVGQHDSESTFVNSCCESPRNHPPQDGLLSAVPNRMEEIIPACTGVVQPLVCHWPSIEKMTN 753
BdMKP1 619 VPHPEKCTGNNS---------TTDGETRLV-----------EYTDSNSEACSCVQLLVYRWPCMEKLTT 667
ZmMKP1.0 629 DFRSEKCTDNSS---------SLHSETRLT-----------ERTDSNSEACSNVQLLVYQWPSMEKLTT 677
ZmMKP1.1 633 EFCSEKCTGNSL---------SSHSETRLI-----------ERTDSNSEVCSNAQLVVYQWPSMGKLTT 681
ZmMKP1.2 633 EFCSEKCTGE---------------EKQ------------------------------LKWCMYG---- 652
CaMKP1.1 703 ATRPGIVDIAGCK--ESSLLRNCVEPSVDSLSRENLKFTSSKGANESGSLQCSKAQTLVYCWPSLEKIDI 770
CaMKP1.2 685 ATRPGIVDIAGCK--ESSLLRNCVEPSVDSLSRENLKFTSSKGANESGSLQCSKAQTLVYCWPSLEKIDI 752
SiMKP1 628 ECCSENCTGNSS---------ISHPETRLT-----------ERTDCNSEDCSNAQLVVYQWPSMEKLTT 676
PtMKP1.0 687 NISLDRVASSDLQHSEASIVNNFDELGKNCHVEEGSGCSMLNGMEESVALSHNIMQPLVCRWPSLERIAA 756
PtMKP1.1 653 NISLVRVASPDLYHKEAST-------------------------ESVEESCKVMQPLVCRWPSLERIAA 696
PeMKP1.0 687 NISLDRVASSDLQHSEASIVNNFDELGKNCHVEEESGRSMLNGMEESVALSHNIMQPLVCRWPSLERIAA 756
PeMKP1.1 681 NISLVRVASPDLYHKEASTVNNFDELGKNCPVGESSGCYVSNGIEESVEESCKVMQPLVCRWPSLERIAA 750
Metal-binding sites
AtMKP1CaMBD 2

```
TMKP1     668 FARKDLDPKTVFIFVAPNASRSAEAVKMVCVWVGGEYECSKG------------VESIDWQQVAGDFLNQ 725
OsMKP1    681 FTRKDLDPKSVLIFVTPEDSRS-EAVKTVHIWVGGEYESSKC------------VDTVDWQQVVGDFFHL 737
AtMKP1    687 LSRAYLDSESVIAIPLPSDAVGETGSRNLYIWIGKSFSLDNNCSLVDSNKAADTVENVDWVQIGESILCQ 756
NtMKP1    759 LASGFFDSRSVYIIVIPDLNLDENSSNSLYIWVGRDVQWKESPNQVINNESMCEDGHVHWEKVGRRFLIQ 828
GrMKP1    754 FTRSDLDSKSAFAIFLPTAAVDENKDRILYFWIGRSFHLEKRSNQLDSSRVVGDREDIDWNQVAYDVLTK 823
BdMKP1    668 FARKDLHPETVFIFVTPDASSSAEAVRTLCIWIGGEYESSKG------------VDTIDWQQVAGDFLNQ 725
ZmMKP1.0  678 FARKDLDSNSVLIFVTSNAIRR-EAVKMVYVWVGGEIESSKS------------VNAVDWQQVTGDFLHR 734
ZmMKP1.1  682 FARKDLDPKSVLIFVTSNAIRRGEAVKMVYVWVGGENESSKS------------VDSVDWQQVTSDFLHL 739
ZmMKP1.2  652 ---------------------------------------------------------------------- 652
CaMKP1.1  771 FGANHLDSEAAFVIFSPN--IHTHAGNVLYFWVGSSFKLDASQVWLDSDRQTSFVGAVDWNRIGRDLIAR 838
CaMKP1.2  753 FGANHLDSEAAFVIFSPN--IHTHAGNVLYFWVGSSFKLDASQVWLDSDRQTSFVGAVDWNRIGRDLIAR 820
SiMKP1    677 FARKDLDPKSVLFFVASNASRR-EAVKMVYVWVGDENESSKS------------DDTVDWQKVTGDFLHL 733
PtMKP1.0  757 LGTGDLDSKSAFVILVPSRGIGRCETRILYFWVGKSFSDEKGLIQLDSGRLLADSEHIDWSQAGHRVLTQ 826
PtMKP1.1  697 LGTSDLDSKTAFAILVPTRGVGRDETRILYFWVGKSFSDEKNMIQLDNNRLLADSEHIYWSQAGYYVLTQ 766
PeMKP1.0  757 LGTSDLDPKSAFVILVPSRGIGRCETRILYFWVGKSFSDEKGLIQLDSGRLLADSEHIYWSQAGHGVLTQ 826
PeMKP1.1  751 LGTSDLDSKTAFAILVPTRGVGRDETRILYFWVGKSFSDEKNMIQLDNNRLLADSEHIYWSQAGYYVLTQ 820
              <----->
           At MKP1 CaMBD 2

TMKP1     726 KGFSNTLPVKVFKEHETEN-LLEVLDAR------ 752
OsMKP1    738 KELGNTLPVKVYKEHETEN-LLEVLNAR------ 764
AtMKP1    757 MDLPKDTPIKIVRESEDQTELLALLSAL------ 784
NtMKP1    829 KGLATSSLVQIVKEGEEPEQLLKHLPCFSLEKTY 862
GrMKP1    824 VGLPNDTPVKIVKEDEEPMEFLMLLRTL------ 851
BdMKP1    726 KGFSNSLPVKIFKEHETDK-LLEVLDAR------ 752
ZmMKP1.0  735 KGLSDALPIKVFKEHETEN-LLELLNFS------ 761
ZmMKP1.1  740 KGLSNVLPVKVFKEHEAEN-LLELLNVS------ 766
ZmMKP1.2  652 ---------------------------------- 652
CaMKP1.1  839 FSLPKNTVTKIVKENEEPQELLALLSSL------ 866
CaMKP1.2  821 FSLPKNTVTKIVKENEEPQELLALLSSL------ 848
SiMKP1    734 KGLSDALPVKVFKEHETEN-LLEVLNVS------ 760
PtMKP1.0  827 MHLRKDVTVKVVKEDEEPAEFLALLSAL------ 854
PtMKP1.1  767 MGLPKDLTIKVVNEDEEPAEFLALLSAL------ 794
PeMKP1.0  827 MHLPKDVTVKVVKEDEEPADFLALLSAL------ 854
```

Fig. S3: Multiple sequence alignment of mitogen-activated protein (MAP) kinase phosphatase 1 (MKP1) of *Triticum turgidum* (TMKP1: ACB05479.1)*; Oryza sativa* (*OsMKP1: BAF46959.1);* *Arabidopsis thaliana* (AtMKP1: AEE79361.1); *Nicotiana tabacum* (NtMKP1: BAD00043.1)*;* *Gossypium raimondii* (GrMKP1: XP_012459204.1); *Brachypodium distachyon* (BdMKP1: XP_003569010.1); *Zea mays* (ZmMKP1.0: XP_008648619.1, ZmMKP1.1: XP_008656547.1,
